# Measurement of urban vitality with time-lapsed street-view images and object-detection for scalable assessment of pedestrian-sidewalk dynamics☆

**DOI:** 10.1016/j.isprsjprs.2025.01.038

**Published:** 2025-03

**Authors:** Ricky Nathvani, Alicia Cavanaugh, Esra Suel, Honor Bixby, Sierra N. Clark, Antje Barbara Metzler, James Nimo, Josephine Bedford Moses, Solomon Baah, Raphael E. Arku, Brian E. Robinson, Jill Baumgartner, James E Bennett, Abeer M. Arif, Ying Long, Samuel Agyei-Mensah, Majid Ezzati

**Affiliations:** a Department of Epidemiology and Biostatistics School of Public Health Imperial College London London UK; b MRC Centre for Environment and Health School of Public Health Imperial College London London UK; c Department of Geography, McGill University Montreal QC Canada; d Centre for Advanced Spatial Analysis, University College London London UK; e Institute of Public Health and Wellbeing, University of Essex Colchester UK; f School of Health & Medical Sciences, City St George’s, University of London; g Science of Cities and Regions Group, Alan Turing Institute London UK; h Department of Physics, University of Ghana Accra Ghana; i Department of Environmental Health Sciences, School of Public Health and Health Sciences, University of Massachusetts Amherst USA; j Department of Equity, Ethics and Policy, School of Population and Global Health, McGill University Montreal QC Canada; k Department of Epidemiology and Biostatistics, School of Population and Global Health, McGill University Montreal QC Canada; l School of Architecture and Hang Lung Center for Real Estate, Key Laboratory of Eco Planning & Green Building, Ministry of Education, Tsinghua University Beijing China; m Department of Geography and Resource Development, University of Ghana Accra Ghana; n Regional Institute for Population Studies, University of Ghana Accra Ghana

**Keywords:** Urban vitality, Street-view images, Object detection, Time series analysis, Imagery analytical, GIS data fusion

## Abstract

Principles of dense, mixed-use environments and pedestrianisation are influential in urban planning practice worldwide. A key outcome espoused by these principles is generating “urban vitality”, the continuous use of street sidewalk infrastructure throughout the day, to promote safety, economic viability and attractiveness of city neighbourhoods. Vitality is hypothesised to arise from a nearby mixture of primary uses, short blocks, density of buildings and population and a diversity in the age and condition of surrounding buildings. To investigate this claim, we use a novel dataset of 2.1 million time-lapsed day and night images at 145 representative locations throughout the city of Accra, Ghana. We developed a measure of urban vitality for each location based on the coefficient of variation in pedestrian volume over time in our images, obtained from counts of people identified using object detection. We also construct measures of “generators of diversity”: mixed-use intensity, building, block and population density, as well as diversity in the age of buildings, using data that are available across multiple cities and perform bivariate and multivariate regressions of our urban vitality measure against variables representing generators of diversity to test the latter’s association with vitality. We find that two or more unique kinds of amenities accessible within a five-minute walk from a given location, as well as the density of buildings (of varying ages and conditions) and short blocks, are associated with more even footfall throughout the day. Our analysis also indicates some potential negative trade-offs from dense and mixed-use neighbourhoods, such as being associated with more continuous road traffic throughout the day. Our methodological approach is scalable and adaptable to different modes of image data capture and can be widely adopted in other cities worldwide.

## 1 Introduction

In urban planning, the concept of urban vitality is characterised by continuous use of street and sidewalk infrastructure in city neighbourhoods throughout the day (Sena, 2017). This in turn produces safe, economically viable, socially cohesive and attractive neighbourhoods (Jacobs, 1961; Lynch, 1981; Montgomery, 1995). Globally practised urban planning principles such as walkable streets, mixed land use (i.e. containing a diversity of commercial, industrial and residential uses) and accessible public spaces are often in pursuit of generating urban vitality (Talen, 2013).

Novel data sources, analytical techniques and computational methods have brought renewed attention to the essential claim that urban vitality is predicated on the density, mixed-use and physical diversity of nearby buildings and roads (Shi et al. 2019; Bibri and Krogstie, 2017) collectively described as urban form. Despite its enduring influence, the empirical relationship between urban vitality and its neighbourhood-level determinants has been difficult to evaluate precisely due to the complex and emergent nature of pedestrian activity patterns. Characterising this relationship is essential for developing data-informed urban design, planning, and administrative approaches across diverse geographic settings.

Studies using mobile phone, GPS location or survey data have investigated urban vitality in the context of Western European countries (De Nadai et al., 2016; Delclòs-Alió et al., 2019; Gómez-Varo et al., 2022; Jacobs-Crisioni et al., 2014; Scepanovic et al., 2021), South Korea (Sung et al., 2015, 2013) and China (Kang et al., 2021; Yue et al., 2017), finding supportive evidence for the relationship between vitality at the level of streets and the surrounding neighbourhood urban form characteristics. However, such studies typically rely on data highly specific to individual cities and regions. Furthermore, the operational definitions of vitality used in each study are highly sensitive to the method of data collection, such as the density of calls from a particular phone network provider, which are not transferable between contexts. This inhibits systematic comparisons between cities and regions for generalisable insights. A scalable, representative and geographically transferable approach to studying the interplay between urban vitality and its corresponding environment is therefore needed.

In particular, significantly less is known about the quantitative relationship between urban vitality and neighbourhood-level characteristics in low- and middle-income country cities, such as those of sub-Saharan Africa (SSA), which experience the fastest urbanisation globally (United Nations et al., 2019) and unique patterns of development (Lall et al., 2017; Taubenböck et al., 2020). Assessing the contextual relevance of urban vitality for SSA cities requires locally relevant data on the dynamics of the human-environment interface. While mobile phone data has seen some application in this regard in SSA cities (Batran et al., 2018; Kung et al., 2014; Wesolowski et al., 2015), there are significant limitations in its capacity to represent and capture the full diversity of city-street users (Blumenstock, 2018; Blumenstock et al., 2015; Wesolowski et al., 2013, 2012). Urban vitality and its proposed benefits were originally described as a largely visual characteristic of city streets. We therefore demonstrate an approach to measuring urban vitality using a time-lapse of images centred on the main thoroughfares of city streets, known as street-view imagery, and the relationship of urban vitality with nearby urban form. We construct an urban vitality index, which can adapt to different frequencies, durations, camera perspectives and coverage of data capture, based on the coefficient of variation in counts of pedestrians detected from images using object detection (Nathvani et al., 2022). This directly measures the constancy of pedestrian presence in the street over time using a source of data that can be captured flexibly and locally from any appropriate set of cameras without the need for complex IT infrastructure or even external power sources (Clark et al., 2020), making it suitable for deployment in a global context.

We demonstrate the utility of this measure in Accra, Ghana using time-lapsed street-view images collected over 15 months between April 2019 to June 2020 at 145 representative urban sites (Clark et al., 2020). Using our index, we use multivariate linear regression to test the widely held hypothesis that vitality, i.e. the constancy of footfall in a given neighbourhood, is related to urban form and design characteristics, based on mixture of nearby uses, density of population and buildings, diversity in the ages of buildings and short blocks (i.e. high density of street intersections). High spatial resolution data to inform these measures are derived from sources available in other African cities, to support the transferability of our approach. In employing this method for Accra, we demonstrate for the first time, the empirical relevance of density and mixed-use for urban vitality in an African city.

## 2 Background, methodological context and contributions

### 2.1 Background

The concept of *urban vitality* was first outlined in Jane Jacobs’ book “Death and Life of Great American Cities”, which highlights the key role streets and sidewalk usage play in the nature of cities and their appeal. Urban vitality is described as continuous pedestrian activity on the sidewalks of a street, which serves to create a safe environment that deters criminal activity, foster social cohesion and trust between users, and assimilate children into their communities (Jacobs, 1961). An often overlooked element of this description of vitality with respect to footfall is that it is relative rather than absolute: “Sheer numbers of people using city streets, and the way those people are spread through the hours of the day, are two different matters … it is important to understand that numbers, in themselves, are not an equivalent for people distributed through time of day.” (Jacobs, 1961).

Therefore, continuity, as opposed solely to large but fluctuating pedestrian footfall, is considered an essential characteristic of vitality (Kang et al., 2021). As well as being relevant for the perceived attractiveness of a neighbourhood, urban vitality and the continuous use of city streets has since been associated with perceived safety (Hillier, 2012; McMillen et al., 2019), physical activity (Gunn et al., 2014; Heath et al., 2006; Wei et al., 2021), economic activity (Martínez et al., 2017; Peña, 1999; Timalsina, 2011), social cohesion (Bjornstrom and Ralston, 2014; Sonta and Jiang, 2023; van den Berg et al., 2017) and health (Braun and Malizia, 2015). However, the conditions that promote vitality within a given neighbourhood may in some cases also exacerbate inequality between privileged and marginalised groups, leading to the displacement of the latter and gentrification (Gómez-Varo et al., 2024).

The most significant factor attributed to generating local urban vitality is i) a mixture of primary uses in the surrounding neighbourhood, or *mixed-use*, predicated on the logic that a broad selection of amenities attracts diverse street users across different hours of the day. Three other neighbourhood-level factors, or “generators of diversity” have also been commonly proposed to foster urban vitality: ii) short blocks, i.e. a density of intersections which induce pedestrians to converge along the same streets and integrates nearby functional uses from surrounding streets, iii) a diversity in the age of buildings, to ensure a mixture of more and less affordable commercial rents which sustain diverse businesses, and iv) spatial density of buildings and population to support demand for diverse goods and services. Various studies have sought to investigate the relationship between vitality and these factors, as outlined in subsection 2.2 below.

### 2.2 Methodological context

The relationship between vitality and neighbourhood-level mixed-use intensity, block length, building diversity and density has received renewed attention in urban analytics studies. An emerging body of work constructs indexes which correspond to urban vitality based on metrics derived from mobile phone, GPS or survey data (De Nadai et al., 2016; Delclòs-Alió et al., 2019; Gómez-Varo et al., 2022; Jacobs-Crisioni et al., 2014; Kang et al., 2021; Scepanovic et al., 2021; Sung et al., 2015, 2013; Yue et al., 2017). In Appendix Table A, we outline a number of such studies along with the data and methods they employ. In particular, indexes constructed to represent vitality typically rely on absolute measures of either observed pedestrians from manual auditing, or from the density of calls or connections from mobile devices.

These vitality indexes are subsequently compared to measures of the four generators of diversity hypothesised to support urban vitality, typically in a regression framework. Common to these studies are a focus on high- and middle-income country cities (Western Europe, South Korea, China), the use of mobile phone data, Shannon entropy as a measure of mixed-use, multivariate linear regression for modelling and a reliance on comprehensive, administrative building footprint data with construction years, business and population information. However, the use of data sources specific to certain cities or countries inhibits a holistically repeatable and transferable method for measuring vitality and its determinants. This has inevitably created a fractured landscape within the literature in which key results, such as the strength of the relationship between vitality and mixed-use, are not directly comparable (Appendix Table A) since they rely on substantially different measures.

Street-view imagery data has emerged as a means of extracting rich, multidimensional information about urban environments (Biljecki et al, 2021; Ibrahim et al, 2020). Visual, in-person auditing of streets has seen application in Shanghai to measure urban vitality and its relationship to nearby urban form (Istrate, 2023). Street-view imagery can be used in a similar manner and, if captured in sufficient quantity, is also amenable to large-scale analysis from image-processing algorithms to extract features relevant to urban vitality (Ma, 2023). In the context of SSA, where a lack of data infrastructure inhibits systematic and quantitative inquiry into urban activity, static street view imagery data captured at spatial representative scales, such as those of Google Street View, has been previously used to map street-level greenery (Liang et al, 2023), the presence of waste and refuse (Umar et al, 2023) and infer mobility patterns (Kago, 2024). However, these works have typically focused on prioritising spatial rather than temporal coverage since sources such as Google Street View are typically updated on an annual basis in the best cases. This leaves a conspicuous gap between the concept of vitality as described in Section 2.1 and the data needed to capture it. In addition, to our knowledge, no studies have sought to use images for inferring urban activity in an African city.

Images repeatedly captured at the same location over time, such as video or time-lapsed imagery, have the potential to address this short-coming, enabling direct observation of urban vitality by analysing the presence of people over time on a given street (Li, H. et al 2022). Our work with street-view time-lapsed imagery advances this emerging field of study in the following ways.

- Our metric of urban vitality is closely aligned with the original conception of urban vitality, using visual change in the occupancy of city streets as seen in street-view images, as opposed to mobile phone, GPS or survey data; all pedestrians within the field of view of the camera are equally considered. This is more appropriate for studying the global context, where phone ownership and usage varies within and between cities.
- The scale of our urban vitality measure is generated at the street level and the extent of spatial buffer in which we consider neighbourhood level determinants is based on intuitive considerations of pedestrian movement, rather than predetermined administrative or aerial units. This enables a flexible, transferable approach which can also determine how spatial extent affects the strength of relationship between vitality and urban form.
- Our sampling approach covers 145 representative locations within the administrative boundary of the same city, capturing 2.1 million images over the course of a year.
- Our measure of mixed-use is based on widely available Open-StreetMap data, which lends itself to transferability in other settings and further enables us to identify specific categories of amenities which may be associated with vitality and mixed-use.
- Our measure of the age of buildings is derived from changes in remote sensing imagery, which is spatially representative and more appropriate for a setting such as SSA, where building development is frequently unplanned and unregistered, and therefore not captured by administrative data sources as in high-income country cities. Since high resolution satellite imagery is routinely captured, such changes are also possible to observe on the global scale at finer temporal intervals (i.e. an annual basis) than typically possible from administrative data.
- Ours is the first study of its kind in sub-Saharan Africa and can be used as a transferable model for further investigations into urban vitality in other SSA cities in the future.

### 2.3 Geographic context

Data used to measure and characterise urban vitality in our study are time-lapsed street-view images captured in the Greater Accra Metropolitan Area (GAMA), the administrative boundary of Ghana’s capital city Accra (Ghana Statistical Service, 2020). In the half-century following independence, Accra has become a hub in SSA for technology, business and education (ARUP and Cities Alliance, 2016; World Bank, 2015), with growth characterised by diverse land use and large spatial variations in ethnicity, wealth, health (Bixby et al., 2022; Clark et al., 2021b), neighbourhood environmental quality (Alli et al., 2023; Clark et al., 2021a) and infrastructure (Adugbila et al., 2023; Ghana Statistical Service, 2020). Public transport is limited (World Bank, 2015), with privately owned and informally operated minibuses known as tro tros commonly used for transport, alongside private vehicle ownership (Birago et al., 2017; Musah et al., 2020). Commercial activity on streets in the form of informal traders and businesses, is a large part of the local economy (Asante and Mills, 2020; Brown et al., 2010) as in many other SSA cities (Ligthelm, 2006; Onyebueke and Geyer, 2011).

Despite an urgent need for new retail spaces, few officially planned commercial units are delivered to meet demand in GAMA, motivating the conversion of significant proportions of residential spaces to commercial use (Gaisie et al., 2019; Oosterbaan et al., 2012). Evidence from Accra (Oosterbaan et al., 2012) indicates that a diversity of new and old buildings produces diversity in rents, with an interplay between residential and commercial units: “Because the properties are typically old and in poor condition, several of the owners indicated that they are eager to rent or sell their units… Other property owners sell part of their properties for conversion and continue to live in the remaining part… … owners admitted that this practice has become a lucrative business activity for them as well as businesses that operate in the converted properties”. We therefore focus on the proportion of all buildings retained over time as seen from remote sensing (described in section 3.3.3) as a measure of building age diversity in our study.

## 3 Methods and data

### 3.1 Imagery derived pedestrian and vehicular data

**Fig. 1:**
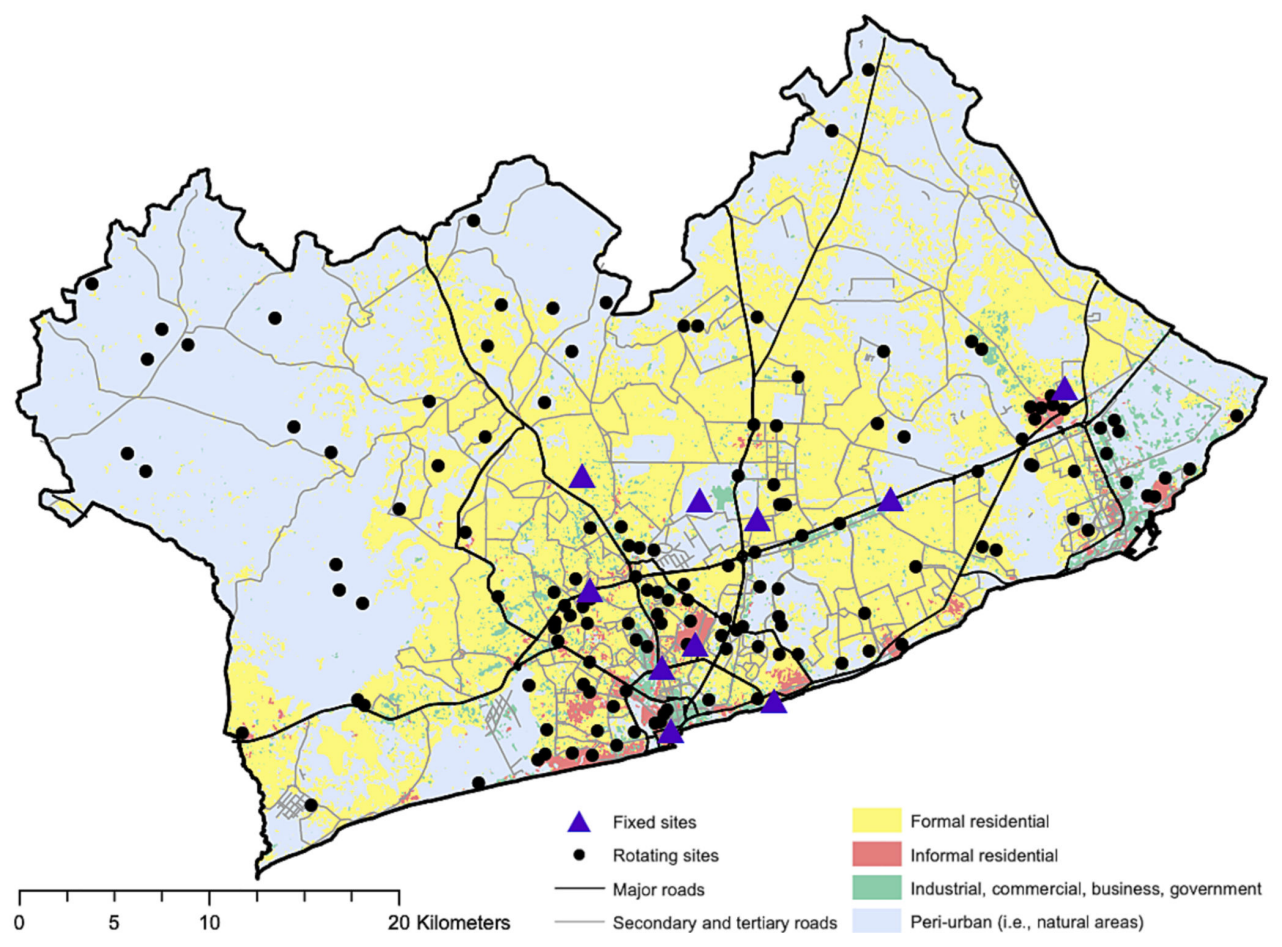
Camera locations.

From April 2019 to June 2020, we captured images at 135 locations throughout GAMA for a period of one week each, with 10 additional fixed sites at which data were collected throughout the 15-month period, shown in Fig. 1. Sites were selected based on a sampling approach which covered low and high traffic areas, sparsely versus built up areas, low and high income neighbourhoods and rapidly changing versus static neighbourhoods with respect to development, validated as compared with census data (Ghana Statistical Service, 2020) and local knowledge. This was achieved using a land use categorisation raster (World Bank, 2014) covering informal and formal residential, industrial/commercial and *peri*-urban areas (shown in Fig. 1), from which stratified random sampling of longitude and latitude was used to identify representative sites, with twice as many sites weighted within the central Accra Metropolitan Area boundary, due to its greater mixture of land categories and population density. Full details may be found in our study protocol paper (Clark et al., 2020).

The locations of our cameras within the GAMA boundary. The different land use classifications (World Bank, 2014) used to stratify the selection of sites is shown, along with the major road network. Figure reproduced from previous work (Clark et al., 2020) by the authors.

At each location Moultrie-M50 camera traps were deployed and positioned at a target height of ∼ 4 m above ground, overlooking the main thoroughfare of the street. Images were captured at five minute intervals with 20-megapixel quality in a 36.7° field of view. Certain measurement sites had two cameras (90 sites) or one (55 sites), based on whether one or two fields of view sufficiently captured the public streetscape. When below a certain threshold of light (after sunset and before sunrise in Accra, 6 pm – 6am) cameras automatically switched to night-vision mode, capturing images in black and white with infrared flash, which we refer to as night-time images. A total of 2.1 million time-stamped images were captured across all sites.

In previous work (Nathvani et al., 2022), we obtained 9.7 million counts of pedestrians and 9.3 million counts of motorised vehicles (a combination of cars, taxis, tro tro, pick-up trucks, motorcycles, lorries and vans) from our images using an object detection algorithm). Examples of detected objects in our images are shown in Appendix Figure A. Counts of people and vehicles detected in each street-view image demonstrated significant spatiotemporal variation within and between sites and form the basis for our measure of urban vitality.

### 3.2 Measure of urban vitality

**Fig. 2:**
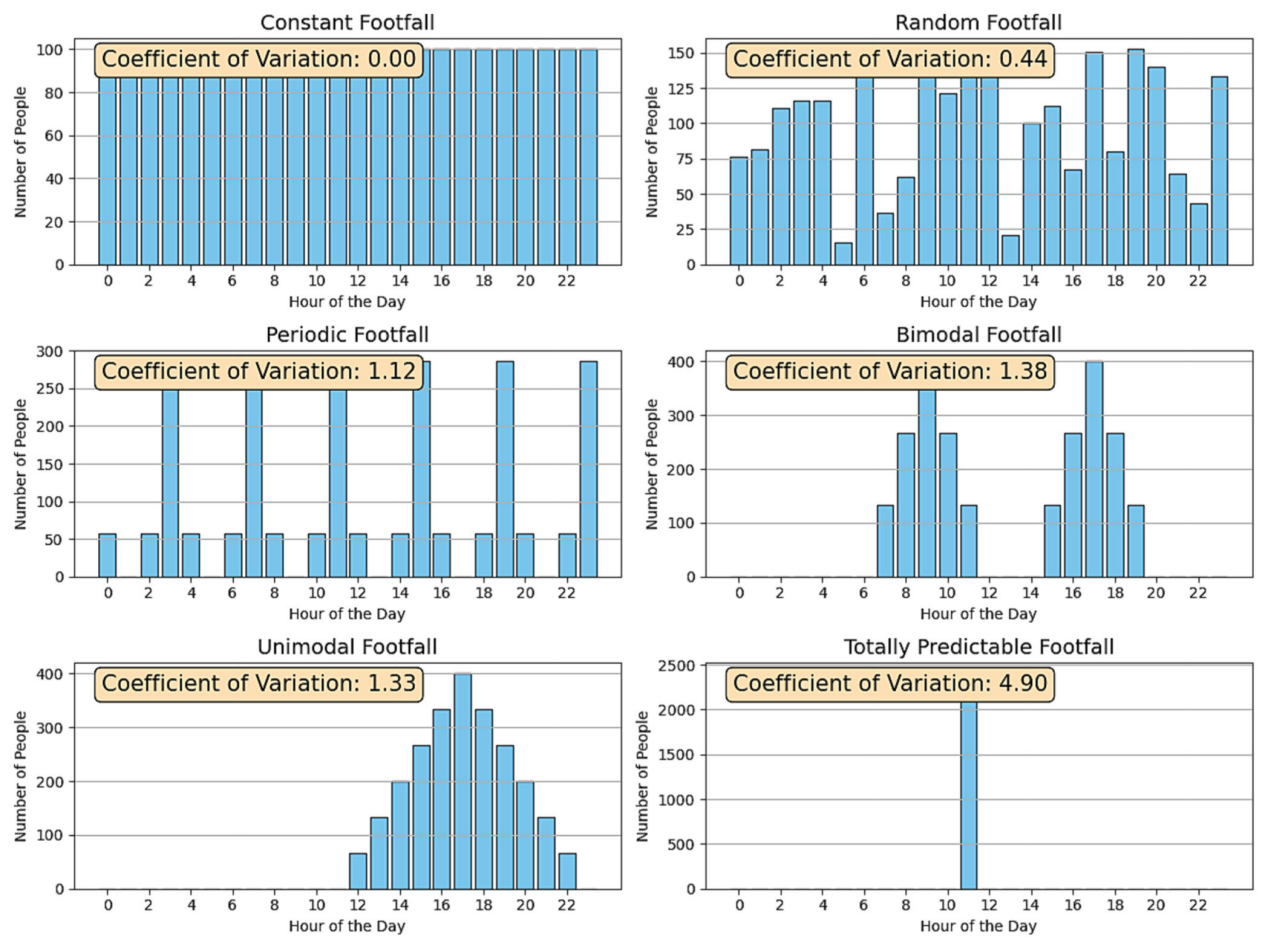
Illustration of coefficient of variation in footfall for different scenarios.

We construct a measure for each site’s urban vitality based on how much the number of people varies throughout the day. Specifically, we calculate the coefficient of variation in the total number of people observed over all hours of the day, based on detected counts of objects labelled as “persons” from our time-lapsed images at each site. Coefficient of variation is suitable because it captures the relative constancy of pedestrian presence, which aligns with the concept of urban vitality as steady footfall throughout the day. Unlike metrics that rely on absolute pedestrian counts or peak-time activity, which could be concentrated to single time periods (e.g. commuter rush hours) and vary greatly across neighbourhoods whilst being highly sensitive to the method of data capture, our measure provides a normalised measure that enables meaningful comparison between sites. This choice allows us to assess vitality consistently across different contexts, making it a robust and transferable metric for evaluating pedestrian dynamics. The coefficient of variation (CV) is defined as the standard deviation (σ) of observed counts across all hours, divided by the mean number of hourly counts (μ). This measure intuitively captures the key idea of constancy of the use of street sidewalks over small periods of time (∼1 h), illustrated in Fig. 2.

Different hypothetical scenarios of distributions in detected pedestrians over time are presented, along with the coefficient of variation calculated for each distribution. It is generally seen that a greater co-efficient of variation corresponds to more irregular and concentrated footfall throughout a given time period (in the extreme, a situation in which all pedestrians are found on the street at a single time of day), whilst regular or sporadic footfall over spread out over greater periods of time correspond to a lower coefficient of variation. In this manner, co-efficient of variation serves as a useful proxy for constancy of footfall throughout the day.

Where the presence of pedestrians is isolated to particular times of day, CV is relatively high, while for perfectly even or uniformly random footfall throughout the day, CV is relatively low. A low CV (more constant of footfall) is considered desirable while greater CV is undesirable due to high variability in footfall. Because CV involves division by the mean it also captures the often overlooked component of the constancy of sidewalk as being relative rather than absolute. This also provides a standardised way to assess urban vitality whilst accounting for small differences in the fields of view of each camera.

In our analysis, we define the average daily coefficient of variation (AD-CV) for day, AD-CV_day_, and night, AD-CV_night_, based on systematic differences within day and nighttime images as described in Section 3.1. The full calculation procedure of these metrics is found in Appendix A. Our primary results are based on an overall daily measure, *AD-CV* = *(AD-CV*_*day*_ + *AD-CV*_*night*_*) / 2*, with sensitivity analyses presented for the individual AD-CV_day_ and AD-CV_night_ measures. Furthermore, in Section 1 we calculate the Pearson correlation coefficient between AD-CV_day_ and AD-CV_night_ across all sites to assess consistency in our measure across the day and night periods latent to our imagery data. Where two cameras exist at a given location we further average across each camera’s independently measured AD-CV to assign a single value for each site. As a measure of validity and consistency, the correspondence between AD-CV measured between two cameras with different fields of view on the same street is also explored in our results by calculating the intraclass correlation coefficient (ICC) of AD-CV between pairs of cameras, as described in Appendix B.

AD-CV has numerous advantages as a measure of urban vitality. It is broadly insensitive to the field of view of the camera; if a given view captures more or less of the thoroughfare of a city street, CV is expected to be reasonably consistent given a flow of pedestrians traversing any given part of it since it normalises by the mean of counts of people (tested in Section 4.1 of our results). Furthermore, AD-CV may be calculated from any source of appropriate time-lapsed street view imagery with sufficient (∼< 1 h) temporal resolution, by obtaining counts of people extracted from each image using widely available and increasingly accurate object detection algorithms (Li and Cao, 2020; Sharma and Mir, 2020). AD-CV may therefore be obtained from existing CCTV and road monitoring networks and is calculable from any number of days (*N* ≥ 1) of observation depending on resources and accessibility. Finally, we can construct identical measures for other object categories whose change over the course of the day may be of interest, such as different types of vehicles. In Section 4.4, we therefore also demonstrate how AD-CV as defined for counts of motorised vehicles detected in the same images as pedestrian counts, relate to generators of diversity.

### 3.3 Measures of surrounding density and diversity

To test the claim that urban vitality, i.e. constancy of side-walk usage, as measured by AD-CV, is related to mixed-use neighbourhoods and other generators of diversity outlined in Section 2, we employ both bivariate and multivariate linear regression. For each independent variable representing generators of diversity, the data used are adopted from sources which are available in other cities.

#### 3.3.1 Mixed-use

**Fig. 3:**
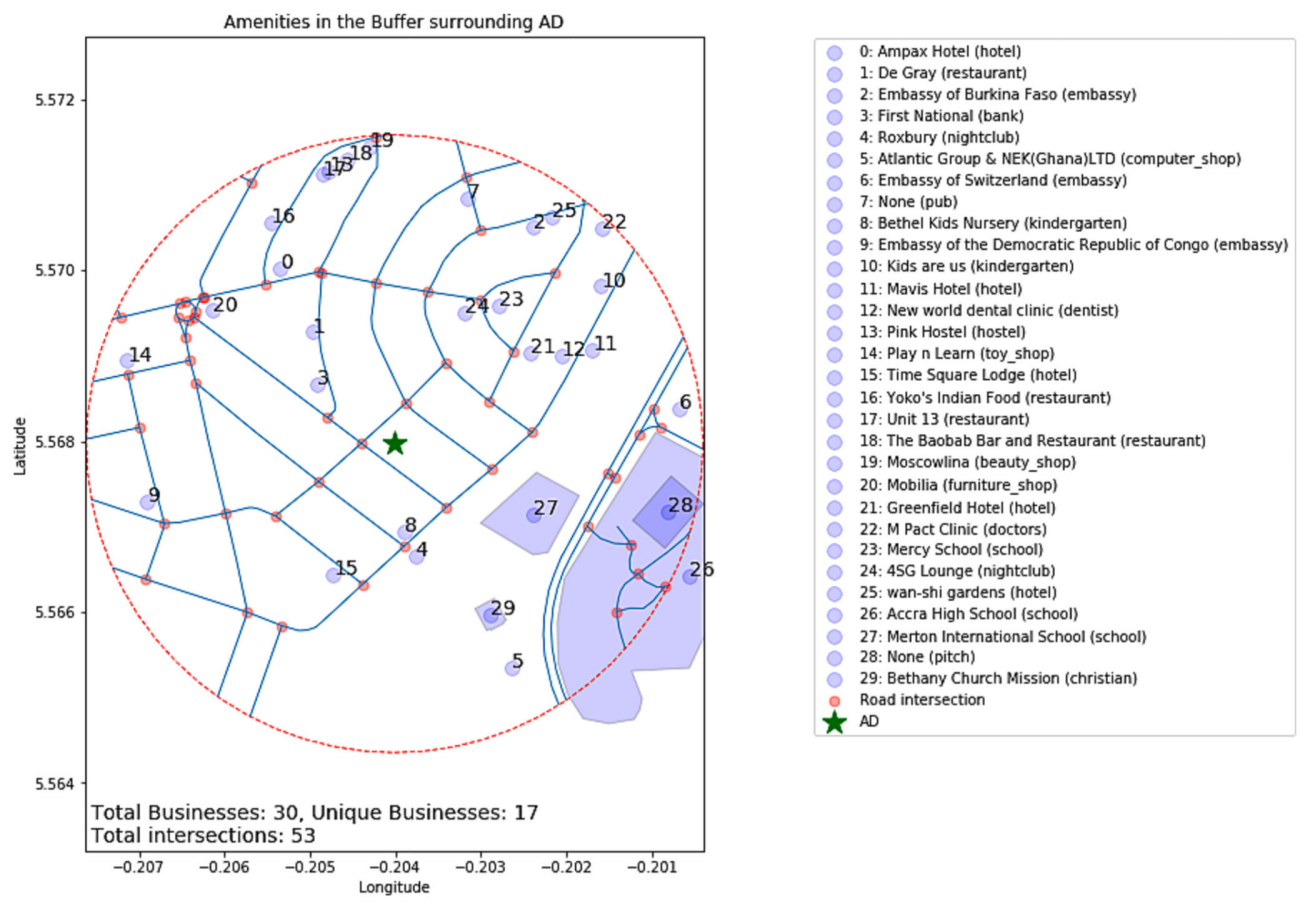
Example camera site with nearby amenities, roads and intersections.

To measure the level of mixed-use in neighbourhoods surrounding camera locations we used point-of-interest (POI) data from Open-StreetMap (OSM) data for 2019, the same year as our images, covering all amenities and places of worship (which are categorised by corresponding faiths) in GAMA. OSM amenities data of this kind is freely available for other SSA cities and globally. Although the coverage of data is estimated to be lower in SSA than other regions, data is being rapidly added in SSA cities and improving in quality (Barrington-Leigh and Millard-Ball, 2017). Data were obtained in the form of points and polygons, each with a corresponding category and name. All categories of amenities and their frequency in our data set are listed in Appendix Table B. We define neighbourhoods as a 400 m radius circular buffer surrounding the camera’s location, corresponding to a five-minute walk in any direction from the camera location. All POI points that fall within this buffer, and any polygon POIs that overlap with this buffer are considered as belonging to the neighbourhood of our camera, as illustrated in Fig. 3.

An example of amenities recorded from OpenStreetMap data (2019) within a 400 m buffer surrounding one of our camera locations (Asylum Down). The total number of amenities, the total number of unique categories and the total number of road intersections within the buffer are all indicated at the bottom of the plot.

The selection of a 400 m radius definition is widely used in urban planning (Azmi and Karim, 2012; Curtis and Olaru, 2010) when considering the availability of amenities accessible from a given point by walking. We test the sensitivity of our analysis to the radius of this buffer in Section 4.5 of our results, re-running analyses with additional buffer sizes, 100 m to 1 km in 100 m intervals.

To measure the diversity of POIs in the buffers around our camera locations, we adopt the metric of Shannon entropy, $H=-{\text{Σ}}_{i=1}^{n}p_{i}\text{log}\left(p_{i}\right)$ where *i* denotes an amenity category and *p*_*i*_ the proportion of the *i*-th category across all amenities within the buffer. This metric is widely used to measure POI and land-use diversity in similar studies (Appendix Table A). Heuristically, one can interpret the Shannon entropy as the uncertainty of correctly predicting a randomly picked amenity’s category membership given the distribution of categories in the buffer, which is more uncertain the more diverse the categories are. To account for the possibility that sheer quantity of amenities rather than diversity may be associated with a lower AD-CV (more constant footfall), we also consider the total number of amenities within the buffer as a control variable in our multivariate linear regression results.

#### 3.3.2 Block density

As well as amenities data, we also obtained road network information from OpenStreetMap for 2019. All roads intersecting with the circular 400 m buffer outlined in 3.3.1 are considered to correspond to a given camera location. To measure street block density, we measure the number of road intersections in the surrounding neighbourhood (i.e. circular buffer), as illustrated in Fig. 3, since smaller block sizes will naturally lead to a greater number of intersections at their vertices for roads of approximately constant width. This definition is also used in comparable studies (Appendix Table A).

#### 3.3.3 Building age

Due to the sparsity of formal planning surrounding the delivery and removal of residential and commercial units in Accra (Arku et al., 2016; Asibey et al., 2023), and corresponding lack of central administrative data on building construction years, we adopt a remote-sensing based approach to estimating the prevalence of older buildings in a neighbourhood. Using high resolution satellite imagery for 2010 and 2019 along with image classification (Nathvani and D., V.,, 2025), as described in Appendix C, we calculate the proportion of existing buildings retained since 2010 in the enumeration area in which a camera is located, as an independent variable in our analysis.

#### 3.3.4 Concentration

To characterise building density, we use the density of buildings per square kilometre as detected by our classification algorithm applied to 2019 satellite imagery (Appendix C), for each census enumeration area in which a camera was located. To estimate population density (residents per square kilometre), we use the most comprehensive spatially resolved estimates currently accessible, which are those from the 2010 census (Ghana Statistical Service, 2020).

#### 3.3.5 Distance from centre

As well as the four generators above, we also include distance from the centre of Accra for each location, in units of kilometers, as a variable in our analysis, since urban vitality has often been associated with dense inner-city environments (Jacobs, 1961; Lynch, 1981; Montgomery, 1995). We defined the reference centre point as the population-weighted, spatial average of centroids of census enumeration areas from the 2010 national Ghana census for GAMA, which corresponds to a location three kilometres west of Kotoka International Airport.

### 3.4 Regression analysis

We frame the hypothesis that diurnal constancy of pedestrian activity on sidewalks, as measured by AD-CV, is predicated on the drivers of diversity outlined in 3.3, in addition to the variable for distance from centre described in 3.3.5, in the form of linear regression analyses as adopted in previous studies (Appendix Table A). In particular, we perform bivariate linear regression between AD-CV and each of the five variables individually and jointly in a multivariate analysis, to assess whether AD-CV and mixed-use are negatively correlated, as anticipated by the planning literature, after accounting for the other factors. For the multivariate analysis, although our data on population density precedes our building density data by nine years it is still highly collinear with it (Pearson R = 0.71) as shown in Appendix Figure B. We therefore employ an ordinary least squares model with all variables included in our primary results, in line with prior studies, and include an equivalent Lasso regression in our sensitivity analysis to identify those variables which may be less relevant for predicting and modelling vitality. Our analysis is descriptive, rather than causal. In the multivariate analysis, all independent variables are Z-scaled (mean of the variable distribution subtracted from each data point and normalised by standard deviation) so that the magnitude of the fit coefficients are comparable across variables. As a further sensitivity measure, although many of our sites are too distant from one another to be meaningfully considered close on the neighbourhood level, for those sites with one or more neighbours within a 1 km spatial buffer of each other (71 of 145) we also produce a spatial lag model, in order to assess potential effects of spatial autocorrelation for vitality. In secondary analyses, we also perform our regressions separately for AD-CV_day_ and AD-CV_night_ to investigate potential differences on the influence of each generator of diversity in both day and nighttime. We also fit a model to the differences between Z-scaled AD-CV_day_ and AD-CV_night_ at each site in order to investigate whether certain features of urban form might distinguish day and night time vitality behaviour. Finally, our AD-CV measure is constructed based on total, half-hourly counts of pedestrians (the totals observed every thirty minutes, as described in Appendix A). In order to evaluate the sensitivity of our results of this choice, we further perform the OLS regression for quarter-hourly, hourly and two-hourly counts of pedestrians in the images and compare coefficient values for each. Ordinary Least Squares Regression is performed using the *Statsmodels* Python library’s OLS function, and Lasso regression performed using LassoCV function in Python’s Sci-kit Learn library with five-fold cross validation.

Of interest is whether particular categories of amenity are associated with greater levels of mixed-use in a given neighbourhood. This may indicate amenities which support, integrate with or generate mixed-use environments in the context of cities like Accra. We therefore calculate which amenities are most commonly found in more diverse neighbourhoods using Pearson correlation. Those amenities with only a small number of total counts (< 5) across all sites are excluded due to low sample size, leaving 46 unique categories for which regressions are performed. Due to this large number, we use Bonferroni correction to our threshold of statistical significance (p < 0.05 → p < 0.001). In addition, we exclude sites with less than two types of amenity since they are functionally incapable of displaying diversity.

We also repeat our bivariate and multivariate regressions for AD-CV, AD-CV_day_ and AD-CV_night_ constructed from counts of motorised vehicles detected in our images throughout the day to identify whether the same generators of diversity that may attract pedestrian footfall may also influence road traffic at the same neighbourhoods.

## 4 Results

### 4.1 Consistency of AD-CV measure

For AD-CV_day_ and AD-CV_night_ at locations with two cameras (each of which provides a separate calculation for AD-CV_day/ night_), we find that the ICC is 0.55 and 0.62 respectively, indicating good agreement for measurements of AD-CV as recorded from cameras pointed in different directions at the same site. Similarly, the Spearman-Brown adjusted reliability obtained across sites is 0.71 and 0.76 for AD-CV_day_ and AD-CV_night_, indicating that averaging observations from different cameras in the manner we have done yields a reliable estimate of AD-CV.

### 4.2 Bivariate and multivariate regression analyses

**Fig. 4:**
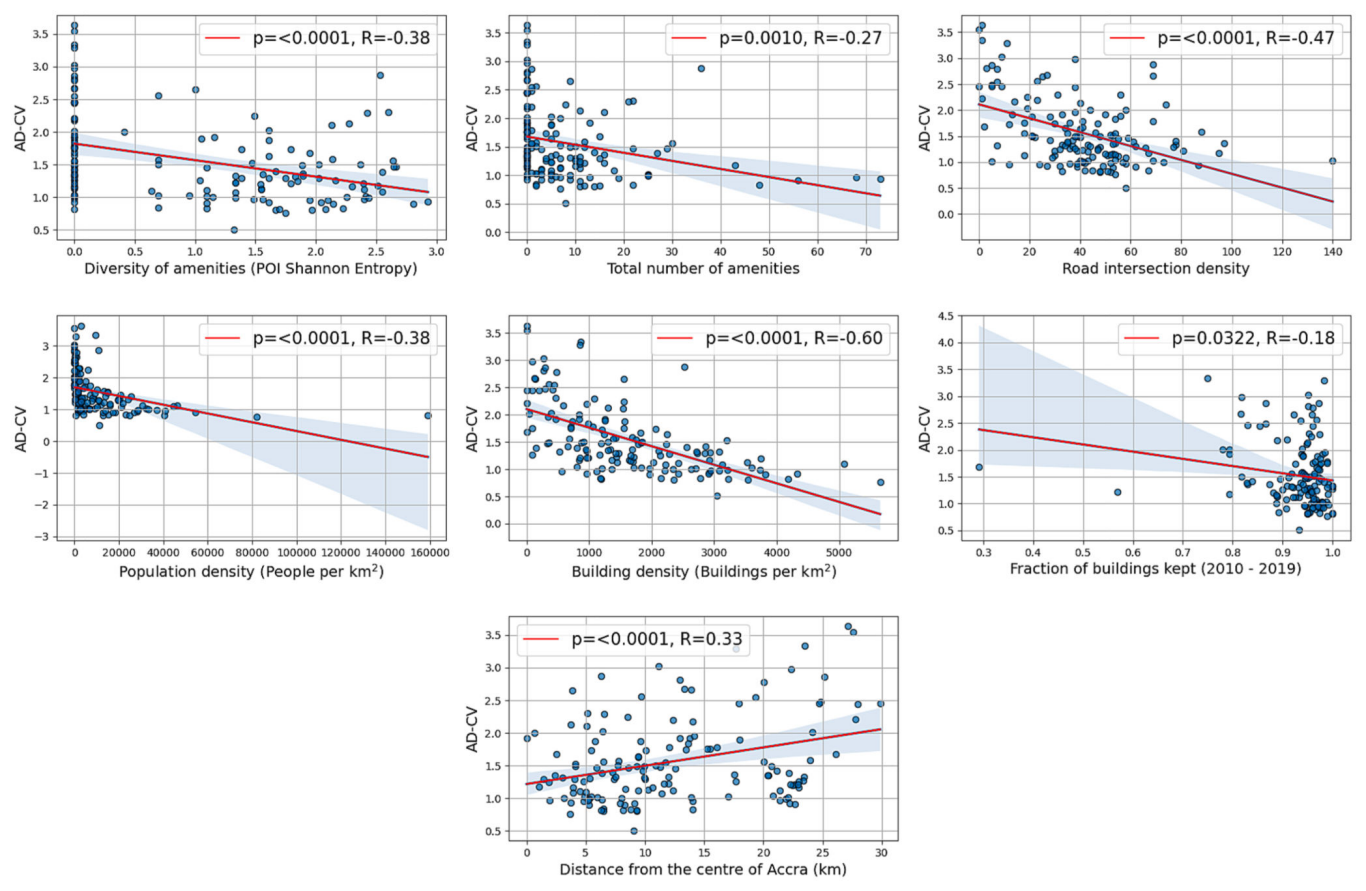
Bivariate linear regressions of AD-CV against generators of diversity.

In bivariate analysis, AD-CV is found to be negatively correlated with POI-based Shannon entropy (R = −0.38, 95 % Confidence Interval (CI) [-0.51, −0.23]), indicating that the constancy of footfall throughout the day is associated with mixed-use environments. However, observing the scatterplot of data (Fig. 4) we find a significant number of locations with zero Shannon entropy due either a lack of any amenities or merely a single amenity, which cannot, definitionally, be diverse. After excluding sites with no amenities, the relationship between AD-CV and mixed-use (POI Shannon entropy) is diminished (R = −0.21, CI = [-0.39, −0.00]), and disappears when considering only sites with two or more amenities (R = 0.03, CI: [-0.18, 0.24]).

Scatterplots for AD-CV against different variables representing generators of diversity, or related variables hypothesised to have an influence on urban vitality. With the exception of Distance from Centre, the hypothesised direction is a negative trend, indicating that a greater magnitude of a given variable is associated with greater constancy of footfall, hence a lower measure of AD-CV.

Bivariate correlation of AD-CV with the other three generators of diversity all indicate relationships in accordance with standard assumptions. Building density (R = −0.62, CI: [−0.70, −0.49]) and road intersections (R = −0.47, CI: [−0.59, −0.33]) are most strongly correlated with lower AD-CV, while population density (R = −0.28, CI: [−0.51, −0.23]) and proportion of buildings retained since 2010 (R = −0.18, CI: [−0.34, −0.02]) follow the expected direction of correlation, albeit to a lesser extent. AD-CV was also positively correlated with distance from the centre of Accra (R = 0.33, CI: [0.17, 0.46]), which follows the expected pattern of greater vitality closer to the urban core.

**Fig. 5:**
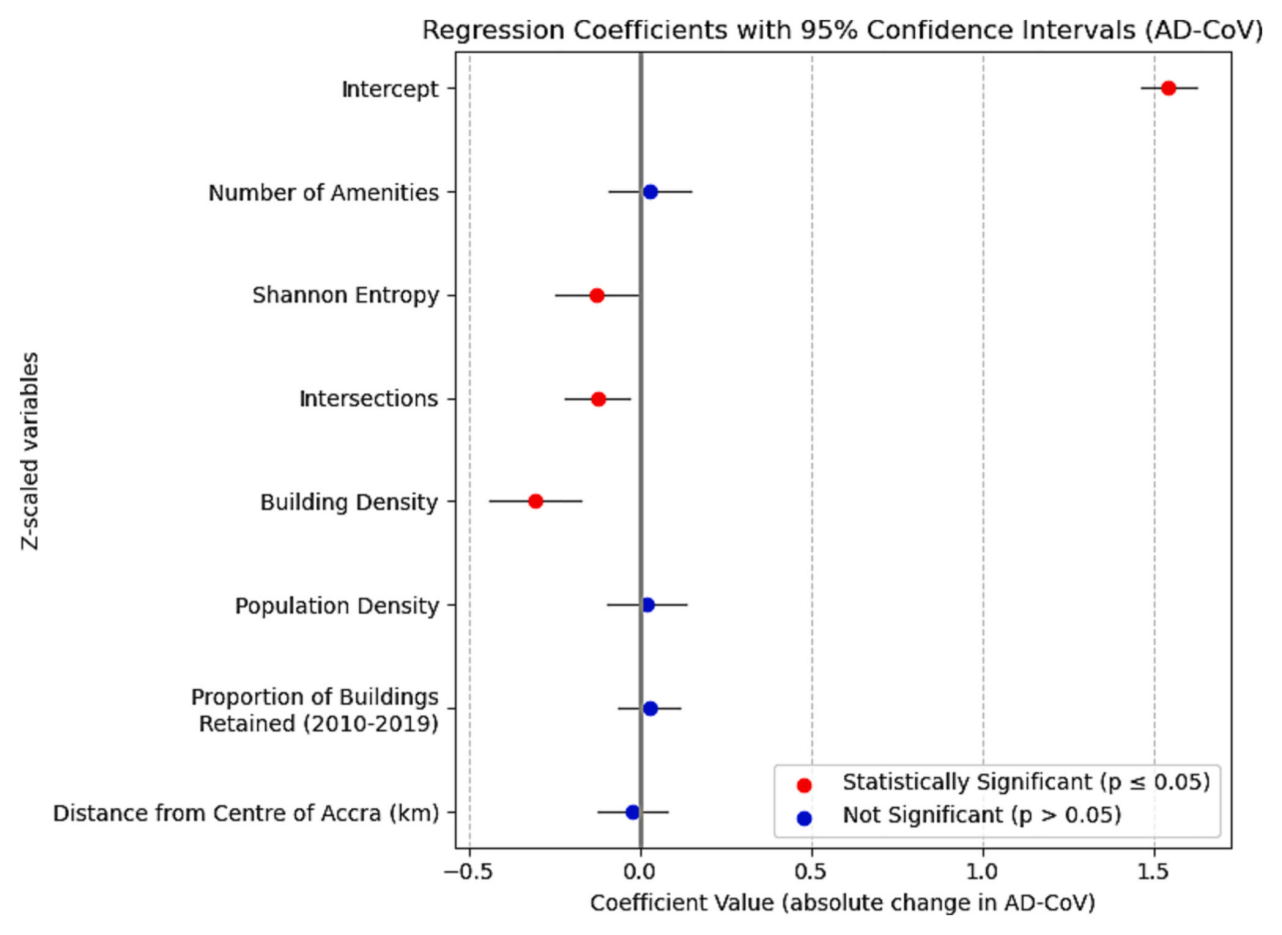
Coefficients from multivariate linear regression of AD-CV against generators of diversity.

In joint, multivariable regression of AD-CV against five Z-scaled variables representing generators of diversity, as well as the total number of amenities and distance from centre as independent control variables, the total explained variance of the linear model (adjusted R^2^) is 38 % (41 % unadjusted). We find that POI Shannon entropy is still independently associated with lower AD-CV (regression coefficient (coef): −0.120, CI = [−0.237, −0.002]), shown in Fig. 5. Intersection (coef: −0.13, CI: [−0.22, −0.03]) and building density (coef: −0.29, CI: [−0.38, −0.19]) are also both seen in the multivariate regression to be independently associated with a reduction of AD-CV, with density having a greater magnitude of Z-scaled coefficient than Shannon entropy (Fig. 5). Performing the same multivariable regression with Lasso instead of ordinary least squares, with five-fold cross validation to determine the optimal penalty hyperparameter, led to the total removal (coefficient shrunk identically to zero) of the variables representing the total number of amenities and population density, further indicating the lack of relevance for these variables to the prediction of AD-CV. Including a spatial-lag component (which accounts for autocorrelation of the dependent variable) in the OLS regression for those sites with at least one other site within a 1 km radius reduces the influence of the coefficient corresponding to intersection density in the model as seen in Appendix Figure C. The coefficient for the spatial lag component itself is not significant, indicating that our primary results, which do not account for spatial autocorrelation, make a valid assumption.

### 4.3 Analysis of particular amenity categories

The following amenity types (of which there are n samples across all sites) are found to be significantly (p < 0.001) associated with more mixed-use neighbourhoods in bivariate regression: restaurants (R = 0.50, n = 74), mobile phone shops (R = 0.45, n = 14), fast food outlets (R = 0.41, n = 26), hotels (R = 0.40), book shops (R = 0.38, n = 8), ATMs (R = 0.38, n = 16), pubs (R = 0.37, n = 29) and hairdressers (R = 0.37, n = 25). In contrast to the full list of amenities observed in our study (Appendix Table B) these eight categories are disproportionately personal, commercial and service-oriented amenities, compared to other common amenity categories more associated with essential and community needs (e.g. schools, hospitals, places of worship, post offices, banks and pharmacies).

### 4.4 Comparisons of day and night AD-CV

AD-CV_day_ and AD-CV_night_ are correlated with each other across sites (Pearson correlation coefficient: R = 0.79). We further performed bivariate regression of AD-CV_day_ and AD-CV_night_ with the individual generators of diversity variables as in section 4.2 and find that both are individually associated with mixed use (POI-based Shannon entropy) in the expected direction: AD-CV_day_ (R = −0.38, CI: [-0.51, −0.23]) and AD-CV_night_ (R = −0.36, CI: [-0.50, −0.21]). The direction and magnitude of bivariate analysis with variables for building density, population density, intersection density and building ages are all similar to trends seen in the primary results, indicating the expected negative correlation of similar magnitude for AD-CV_day_ and AD-CV_night_ as seen in Appendix Figures D and E.

When comparing the multivariate regression results as performed in Section 4.2 for AD-CV_day_ and AD-CV_night_, we find that POI Shannon entropy is still independently associated with lower AD-CV_day_ (coef: −0.08, CI: [-0.14, −0.01]), but is less certain for AD-CV_night_ (coef: -0.18, CI: [-0.35, 0.02]) as seen in Appendix Figure F. The coefficient for intersection density is less relevant in the multivariate regression for AD-CV_day_ (coef: −0.04, CI: [-0.09, 0.02]) than for AD-CV_night_ (coef: −0.21, CI: [-0.36, −0.06]), suggesting it may play a greater potential role in nighttime vitality than daytime vitality. Building density is seen as a significant variable for both though the magnitude for the corresponding coefficient in the instance of AD-CV_night_ (−0.48) is three times greater than for AD-CV_day_ (−0.13). In order to study whether relative differences between day and nighttime vitality might be influenced by surrounding urban form, we also fit a multivariate model with the same independent variables to the difference between Z-scaled AD-CV_day_ and AD-CV_night_ at each site. However, the model achieved a poor fit (R^2^ =0.038, ${\text{R}}_{\text{adj}}^{2}=-0.013$), with all coefficients broadly centred on zero.

### 4.5 Vehicle AD-CV

As well as pedestrian count derived AD-CV, we considered AD-CV derived from counts of any type of motorised vehicles detected as objects from our images. In Appendix Figure G we show the spatial distribution of both pedestrian and vehicular-based AD-CV for day, night and an average of both. Our pedestrian AD-CV and vehicular AD-CV are both found to be highly correlated between sites (R = 0.75, CI: [0.66, 0.81]), and separately for day (R = 0.74, CI: [0.65, 0.80]) and night (R = 0.73, CI: [0.64, 0.80]). Urban vitality, as envisaged by sidewalk usage, appears to accompany an equivalent activity in road usage by motorised vehicles in Accra, an observation inaccessible from previous comparable studies. Therefore, in bivariate regression, vehicular AD-CV follows the same trends as pedestrian AD-CV with even lower Pearson correlations (i.e. greater magnitude of correspondence) amongst variables representing generators of diversity, with the exception of building and intersections density which are of similar magnitude (Appendix Figure H).

**Fig. 6:**
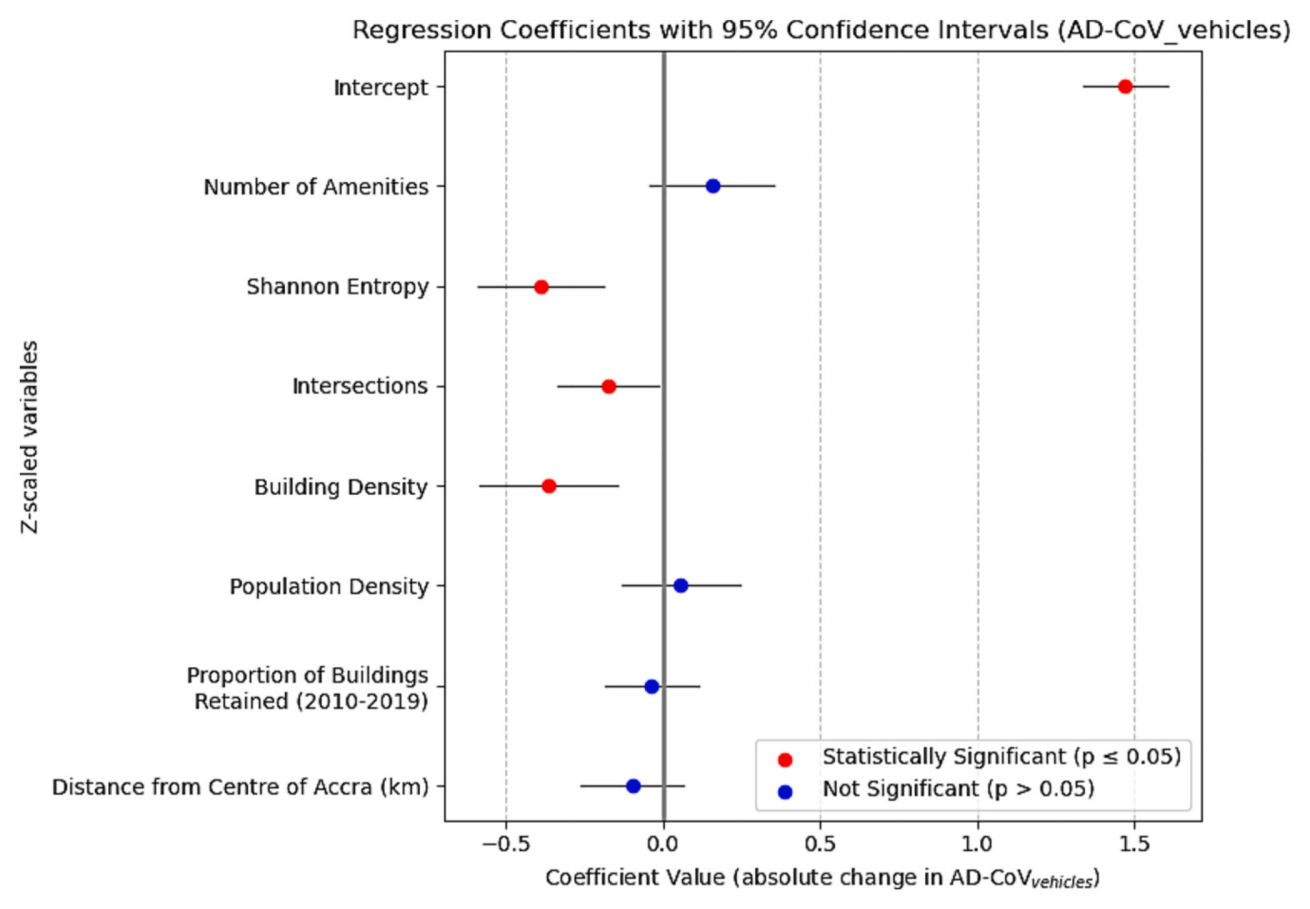
Coefficients from multivariate linear regression of vehicular AD-CV against generators of diversity.

Analogous trends are observed in the multivariate analysis of vehicular AD-CV against all four generators of diversity and total number of amenities, which achieves an adjusted R^2^ of 31 %. Results are similar to that of pedestrian AD-CV except that the magnitude of the coefficient related to Shannon entropy is greater in absolute magnitude (more so than building density), as seen in Fig. 6. In summary, we find evidence that the potential effects of mixed-use neighbourhoods on creating a continued presence of traffic throughout the day may be more influential than its effects on pedestrian footfall in neighbourhoods of Accra.

### 4.6 Buffer size sensitivity

**Fig. 7:**
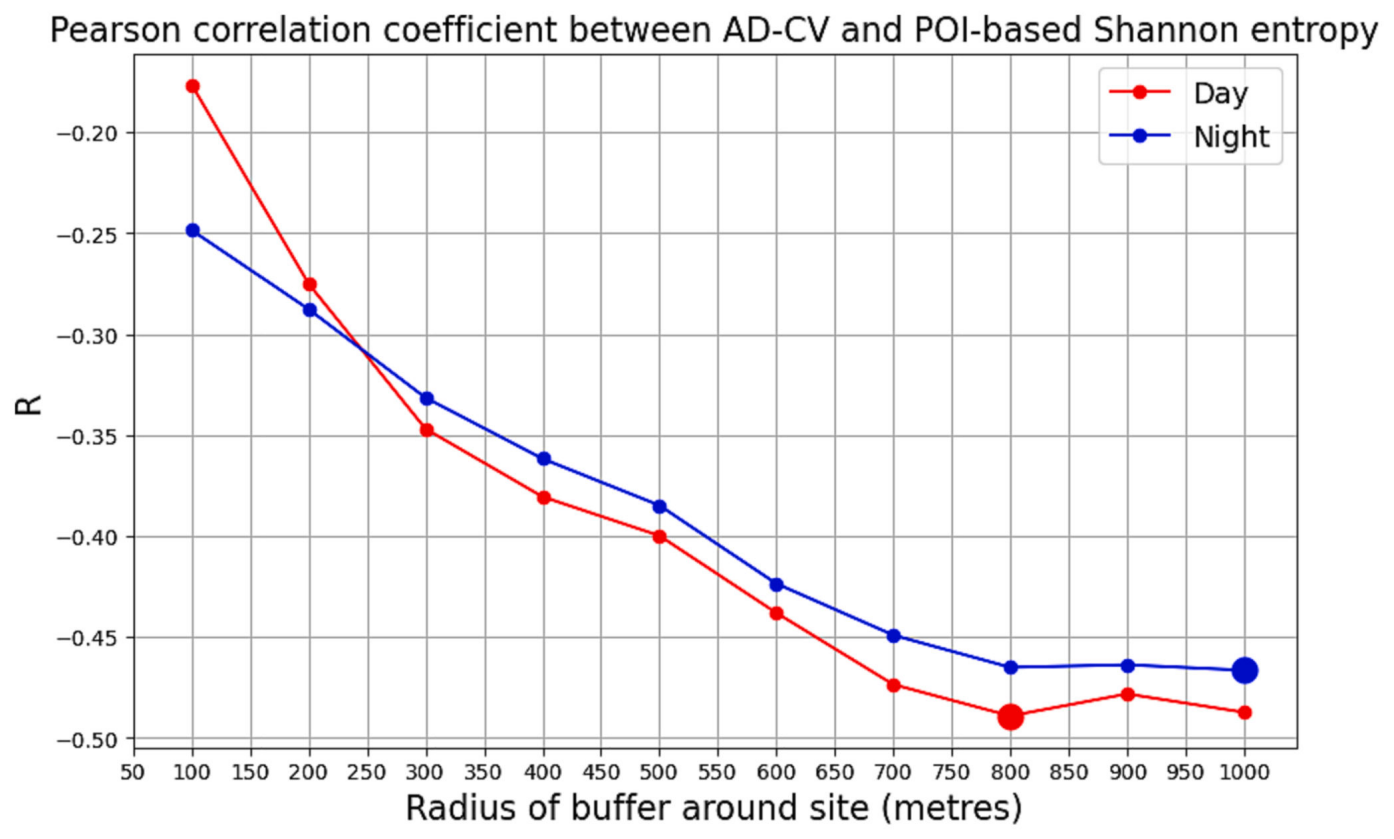
Pearson correlation coefficient of AD-CV and POI-based Shannon entropy (amenity diversity) as a function of buffer size.

Our primary results adopt a definition of neighbourhood consisting of a 400 m circular buffer surrounding a point, based on the widely used heuristic of the distance traversed in a five-minute walk. In order to test the sensitivity of our results to the choice of this buffer, we conducted a series of bivariate Pearson correlations between POI-based Shannon entropy and AD-CV_day_ and AD-CV_night_ with buffers ranging from 100 m to 1 km in 100 m intervals. As seen in Fig. 7, the Pearson correlation increases further in the negative direction (i.e. a greater overall magnitude of correlation) with increasing buffer sizes, up to 800 m for AD-CV_day_ and 1 km for AD-CV_night_. This suggests that when considering the influence of nearby amenities on pedestrian footfall at a given location, a greater spatial extent than usually considered (10-minute walk) may be valid for considering neighbourhood impacts on street level activity, for cities like Accra.

### 4.7 Temporal resolution sensitivity

**Fig. 8:**
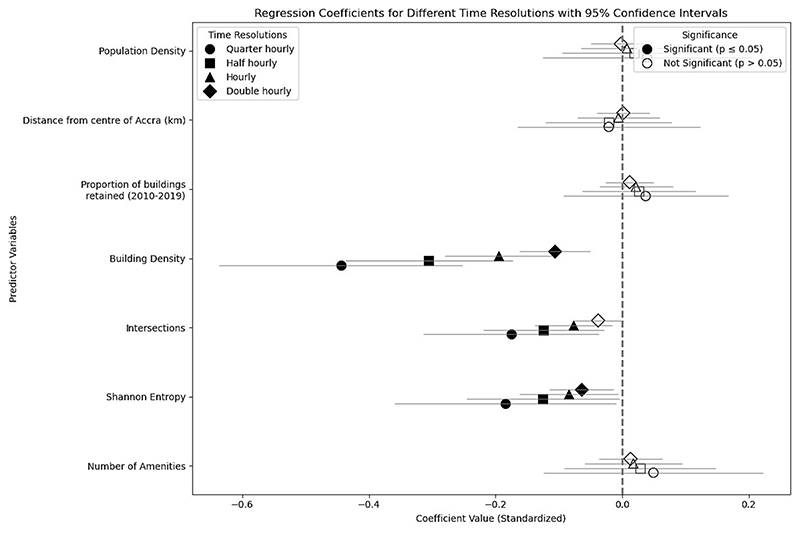
Coefficients from multivariate linear regression of AD-CV, constructed based on different time-intervals, against generators of diversity.

When constructing our AD-CV measure based on different time intervals (the total number of pedestrians recorded in either quarter-, half-, single or two hour intervals) and performing the same multivariate regression for AD-CV in Section 4.2, we find that the shorter the time intervals used to construct AD-CV (as described in Appendix A), the greater the magnitude of the coefficients for building density, intersection density and POI-based Shannon Entropy, albeit with larger confidence intervals, seen in Fig. 8. This indicates that the time-scale on which we consider “constancy” of footfall as a measure for urban vitality may be shorter than typically considered, potentially driven by urban form factors on scales as short as a quarter of an hour.

## 5 Conclusion and discussions

Time-lapsed street-view images captured at sub-hourly, hourly or two-hourly intervals are capable of measuring and monitoring urban vitality. By using changes in the number of people detected in each image from object detection, as embodied in the metric of coefficient of variation, “evenness” of footfall throughout different times of the day is captured visually in a self-consistent and reliable way at different locations in the same city. Our metric of AD-CV captures these characteristics in Accra and can be replicated using any camera capable of capturing time-lapsed images, including CCTV networks being widely deployed in African cities (Jili, 2022; Palinwinde Jacobs, 2021), provided appropriate safeguards are in place for data collection and anonymity. Developments in edge computing methods and advances in object detection algorithms may allow such metrics to be calculated on site without the need for storing potentially sensitive imagery (Huang et al., 2022; Ren et al., 2018). In doing so, a number of conceptual and practical disadvantages associated with other forms of data, such as surveys and mobile phone data outlined in Section 2, may be superseded.

When using AD-CV to empirically investigate urban planning claims on the nature of neighbourhood function and form to attract people at different times of day, we find evidence consistent with its assertions in the context of Accra. In particular, two or more unique kinds of amenities accessible within a five to ten minute walk from a given location, as well as the density of buildings (of varying ages and conditions) and short blocks, are associated with more stable footfall throughout the day. To guide urban planning, high and low CV values may suggest practical interventions. Areas with low CV, indicating consistent pedestrian presence, may be suitable for investment in pedestrian-friendly infrastructure to better support existing vitality. In contrast, areas with high CV, showing variable pedestrian presence, could benefit from targeted interventions, such as encouraging mixed-use development, densification of infrastructure or improving access to transit options. These approaches may increase the constancy of footfall by promoting neighbourhood features that attract and retain diverse users throughout the day.

In the context of SSA cities, where informal trade and diverse socioeconomic settings shape urban life, mixed-use environments support social cohesion, safety, and economic opportunities. Studies on SSA cities have noted the impact of informal commerce on public spaces, underscoring how a variety of street-level activities can contribute to vitality (Asante and Mills, 2020; Brown et al., 2010). However, as seen in Accra, mixed-use settings may inadvertently increase vehicular traffic, raising concerns for air quality, noise pollution, and pedestrian safety. Urban planners may consider design strategies, such as integrated public transit systems and pedestrian-focused infrastructure, to balance vitality and manage traffic impact (Birago et al., 2017; Musah et al., 2020).

Despite their potential benefits, our analysis also indicates potential negative trade-offs for dense, mixed-use neighbourhoods in cultivating urban vitality. In addition to hosting a greater number of detrimental amenities such as fast food outlets, we find evidence that mixed-use neighbourhoods may also be associated with road traffic throughout the day. Despite vitality’s role in promoting safety, more deleterious amenities and more traffic may have a negative impact on the health of those who live, work and play in the neighbourhood, particularly from hazardous environmental exposures such as air and noise pollution in the context of Accra (Alli et al., 2023; Clark et al., 2021a; Wang et al., 2024). This highlights the need for improved pedestrian infrastructure and public transport options, integrated with traffic management policies that cultivate safe street environments (Birago et al., 2017; Musah et al., 2020), which are an essential part of life for many residents in African cities. Such interventions can help to balance urban vitality with reduced traffic congestion and support neighbourhood safety and accessibility goals. Our findings underscore the importance of integrated urban planning approaches that consider both pedestrian and traffic dynamics to foster sustainable, lively, and healthy urban spaces.

Furthermore, mixed-use environments in the context of Accra are likely to include many mobile traders and vendors not captured in OSM data but whose ability to sell goods and services as a vital part of city street commerce (Brown et al., 2010; Skinner, 2008), should be accounted for in street infrastructure and development. In addition, the coverage and consistency of OSM data has not been exhaustively well validated in the context of African cities, with some work showing that the quality of coverage varies considerably depending on the extent of settlement informality (Yeboah et al, 2021). However, similar to the approach we have taken using remote sensing images to infer building footprints, ongoing work aims to improve coverage aided by machine learning techniques (Li, H. et al 2022). Likewise, the ability to extract road network, building and population density information has become increasingly amenable to analysis from remote sensing data even where administrative data is sparse, enabling a consistent approach to quantifying urban form characteristics relevant to vitality in diverse contexts (Metzler et al, 2023). Future work can also contextualise vitality as measured in this work by combining measurements with questionnaires and community engagement surveys to directly assess what amenities, commercial opportunities or opportunities for social contact draw people to a given area across different times of day (Anthony, 2023; Gearin. and Hurt. 2024).

Although we validate the internal consistency of AD-CV as an index, our analysis is descriptive rather than causal, capturing associations in a snapshot in time. Despite availability of images in later years, changes in the categorisations of amenities used by OSM over time meant that our metrics of POI-based Shannon entropy were inconsistent between years, preventing us from using more causal analytical methods for determining the influence of generators of diversity on reducing AD-CV. This may be addressed in future work by retrospectively re-coding amenity types for consistency over time, enabling an analysis of whether changes in the level of mixed-use (the diversity of amenities) precedes a decrease in AD-CV over time as neighbourhoods evolve or vice-versa. Validating the coverage and bias in the availability of amenity categories in OSM data, however, remains a challenging issue, which may be mitigated in future work through comparison to alternative sources of POI data, such as that available from Google.

To our knowledge, this study represents the first attempt to empirically measure urban vitality and its determinants in an African context, where the developmental trajectory of cities is unique in both character and pace. We empirically demonstrate the role mixed-use neighbourhoods may play in supporting urban vitality, contributing to evidence needed to support land-use administration in SSA cities (Du Plessis, 2015; Zelelew and Mamo, 2023). Our methods for measuring urban form factors related to mixed-use are constructed on data available in other African cities and globally, allowing inter-city and regional comparisons to be made for how neighbourhood characteristics affect urban vitality. Given the significance of street activity to SSA cities, image-based AD-CV based analyses of urban vitality may provide a means to generate much-needed evidence for fostering and developing economically productive and lively city neighbourhoods based on planning and design initiatives.

## Supplementary Material

Appendix A. Supplementary data
