# Supplementary material for: Measurement of urban vitality with time-lapsed street-view images and object-detection for scalable assessment of pedestrian-sidewalk dynamics
Source: ISPRS J Photogramm Remote Sens. Author manuscript; Available in PMC 2025 Mar 1. (PMC7617441; doi:10.1016/j.isprsjprs.2025.01.038)
Supplement: Appendix A. Supplementary data [file EMS203319-supplement-Appendix_A__Supplementary_data.pdf]

**Measurement of urban vitality with time-lapsed street-view images and object-detection for scalable assessment of pedestrian-sidewalk dynamics: APPENDICES**

Ricky Nathvani*[1,2], Alicia Cavanaugh[3], Esra Suel[4], Honor Bixby[5], Sierra N. Clark[6], Antje Barbara Metzler[7], James Nimo[8], Josephine Bedford Moses[8], Solomon Baah[8], Raphael E. Arku[9], Brian E. Robinson[3], Jill Baumgartner[10,11], James E Bennett[1,2], Abeer Arif[1,2], Ying Long[12], Samuel Agyei-Mensah[13], Majid Ezzati†[1,2,14]

* First and corresponding author: r.nathvani@imperial.ac.uk, Michael Uren Hub Building, 86 Wood Lane, London, United Kingdom, W12 0BZ

† Senior author

1 Department of Epidemiology and Biostatistics, School of Public Health, Imperial College London, London, UK

2 MRC Centre for Environment and Health, School of Public Health, Imperial College London, London, UK

3 Department of Geography, McGill University, Montreal, QC, Canada

4 Centre for Advanced Spatial Analysis, University College London, London, UK

5 Institute of Public Health and Wellbeing, University of Essex, Colchester, UK

6 Population Health Research Institute, St George's, University of London, London, UK

7 Science of Cities and Regions group, Alan Turing Institute, London, UK

8 Department of Physics, University of Ghana, Accra, Ghana

9 Department of Environmental Health Sciences, School of Public Health and Health Sciences, University of Massachusetts, Amherst, USA

10 Department of Equity, Ethics and Policy, School of Population and Global Health, McGill University, Montreal, QC, Canada

11 Department of Epidemiology and Biostatistics, School of Population and Global Health, McGill University, Montreal, QC, Canada

12 School of Architecture and Hang Lung Center for Real Estate, Key Laboratory of Eco Planning & Green Building, Ministry of Education, Tsinghua University, Beijing, China

13 Department of Geography and Resource Development, University of Ghana, Accra, Ghana

14 Regional Institute for Population Studies, University of Ghana, Accra, Ghana

## Appendix A: Calculation of Average Daily Coefficient of Variation

To calculate our average daily coefficient of variation (AD-CV) metric, we first add up counts of people, as detected from object detection applied to our images, observed every 30 minutes (summed counts of objects every 6 images), to obtain half-hourly counts, then calculate the coefficient of variation over this distribution for each day, and finally average over all days of observation in order to obtain average daily coefficient of variation (AD-CV) for each site. One adjustment is made based on the limitations of our images, as our previous work (Nathvani et al., 2022) indicated that the object detection algorithm we used was sensitive to the change from colour daytime images to grayscale nighttime images taken in darker conditions. We therefore first construct a day and nighttime vitality measure separately for these periods to account for differences in counts that may arise from changes in visibility, rather than true changes to the number of people. Our measures are defined as $\text{AD-CV}_{\text{day}} = \frac{1}{N}\sum_{d=1}^{N} \text{CV}_d$, where $\text{CV}_d = \frac{\sigma_d}{\mu_d}$ is the Coefficient of Variation of each day, *d,* of which there are *N* days of observation. $\sigma_d$ is the standard deviation of half-hourly counts and $\mu_d$ is the mean of half-hourly counts during the day (6am - 6pm). Similarly $\text{AD-CV}_{\text{night}} = \frac{1}{N}\sum_{n=1}^{N} \text{CV}_n$, where *n* denotes individual nights of observation.

**Appendix B: Consistency of AD-CV metric**

Where two cameras exist at a given location we further average across each camera's independently measured AD-CV to assign a single value for each site. The correspondence between AD-CV measured between two cameras with different fields of view on the same street is also explored in our results by calculating the intraclass correlation coefficient (ICC) of AD-CV between pairs of cameras. In intuitive terms, ICC aims to measure whether the variation in calculated AD-CV from cameras at the same site is less than the variation across all sites. A larger ICC corresponds to high intraclass correlation, indicating that AD-CV is meaningfully invariant to camera positioning. We calculate this using the Pinguion Python library's intraclass_corr function (output ICC1; one-way random effects). Similarly, we calculate the reliability of average ratings obtained from both cameras based on Spearman-Brown adjusted reliability which modifies ICC to consider the extent to which averages of measurements are reliable inferences, rather than intra-class measurement agreement (ICC1k in Pinguoin's intraclass_corr function), to assess whether averaging across cameras, as we have done, is consistent.

## Appendix C: Building age measure

In separate work (Nathvani and D., 2025), we used Maxar building footprints for GAMA generated from ~0.3m/pixel resolution satellite imagery for 2019 (Maxar, 2020), to fine-tune Resnet-50 image classification algorithms (He et al., 2016) to detect the presence of buildings or their absence (i.e. empty land) in satellite image tiles corresponding to every building footprint. Satellite images and building footprints of this kind also exist for other SSA cities (Maxar, 2020), from a variety of sources (Microsoft, 2023; Sirko et al., 2021). Our classification algorithm was fine-tuned on 5500 manually labelled satellite image tiles from corresponding footprint locations in both 2010 and 2019 in GAMA (which either contained buildings, or were empty), which was then applied to all building footprint locations for both years. Details on our algorithms' performance are given in Appendix Table C. In this manner, we estimated the proportion of buildings that were either added or removed by 2019, relative to existing building stock in 2010 for each census enumeration area.

**Appendix Figure A: Objects identified in example images.**

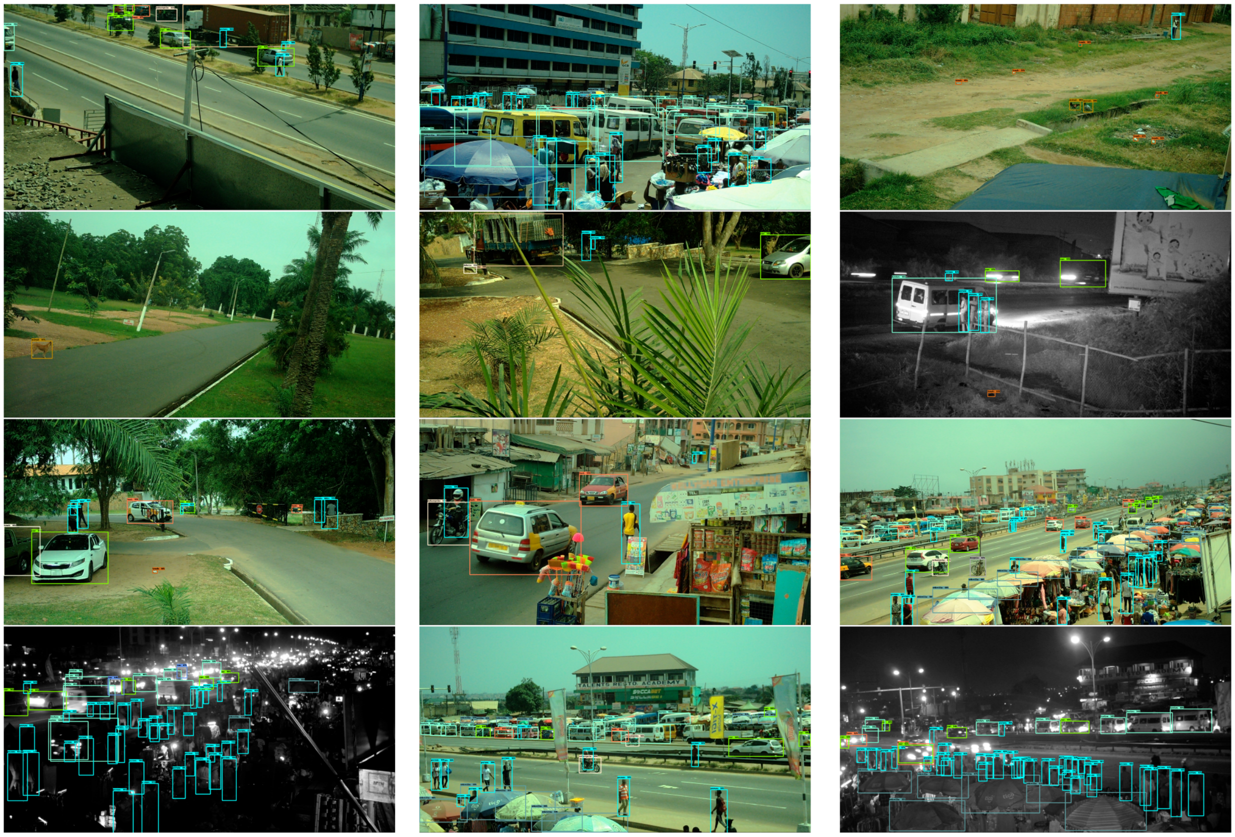

Each identified object is bounded by a box, coloured by object type. The number next to the object names shows the final layer's activation score for the given object's classifier, which may be heuristically interpreted as the network's confidence score in its prediction. "Truck" refers to pick-up truck, "Bowl" refers to cooking bowl/pot, "Stall" refers to market stalls and "Stove" to cookstoves of the variety found in markets (figure reproduced from previous work (Nathvani et al., 2022), by the authors).

**Appendix Figure B: Correlations between urban form factors (generators of diversity)**

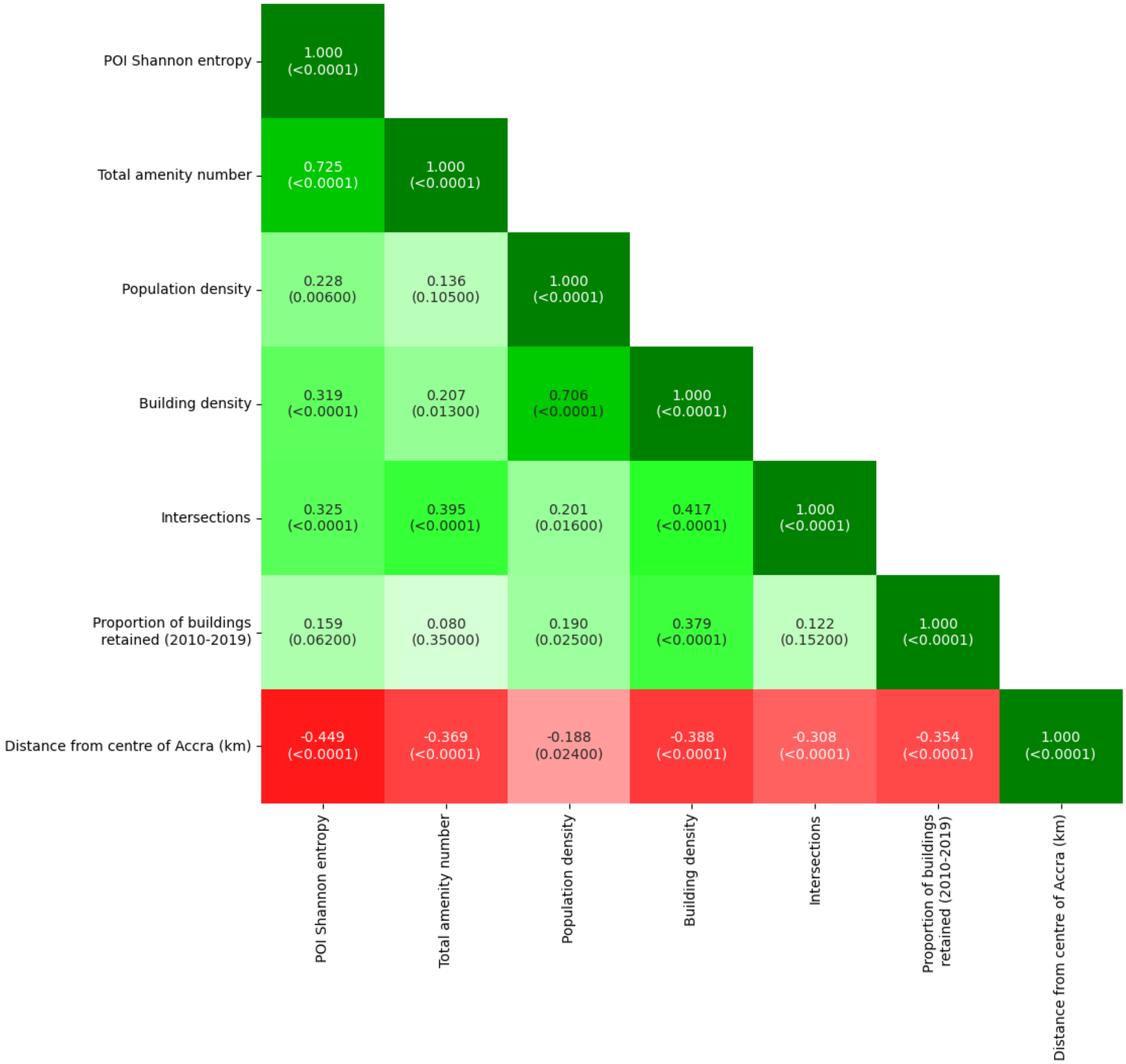


Pearson correlation coefficients between different independent variables constructed to represent generators of diversity in our dataset, as well as the variable for total number of amenities. P values are given for each coefficient in the parentheses below each stated value.

**Appendix Figure C: Coefficients from multivariate Spatial Lag model regression of AD-CV against generators of diversity**

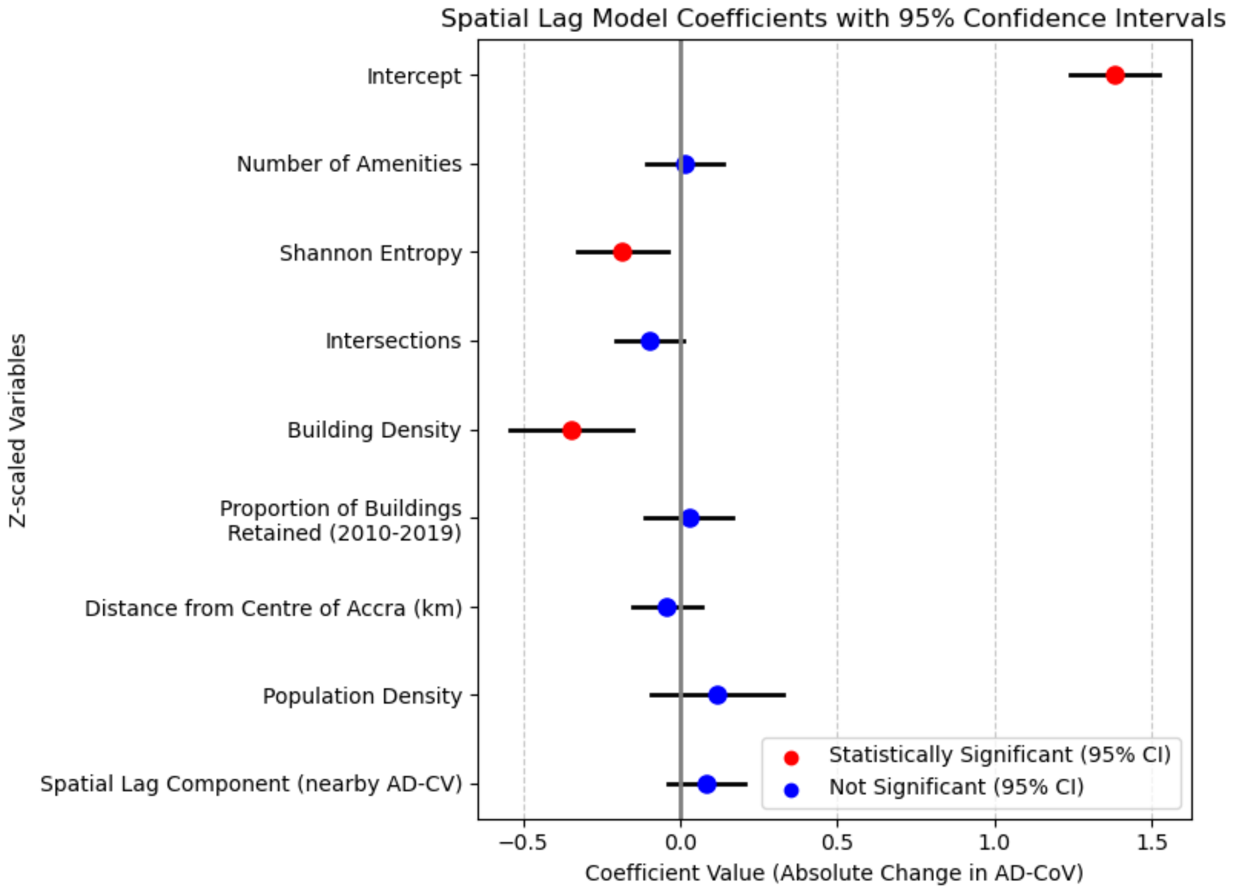

## Appendix Figure D: Single variable linear regressions of AD-$CV_{day}$ against generators of diversity

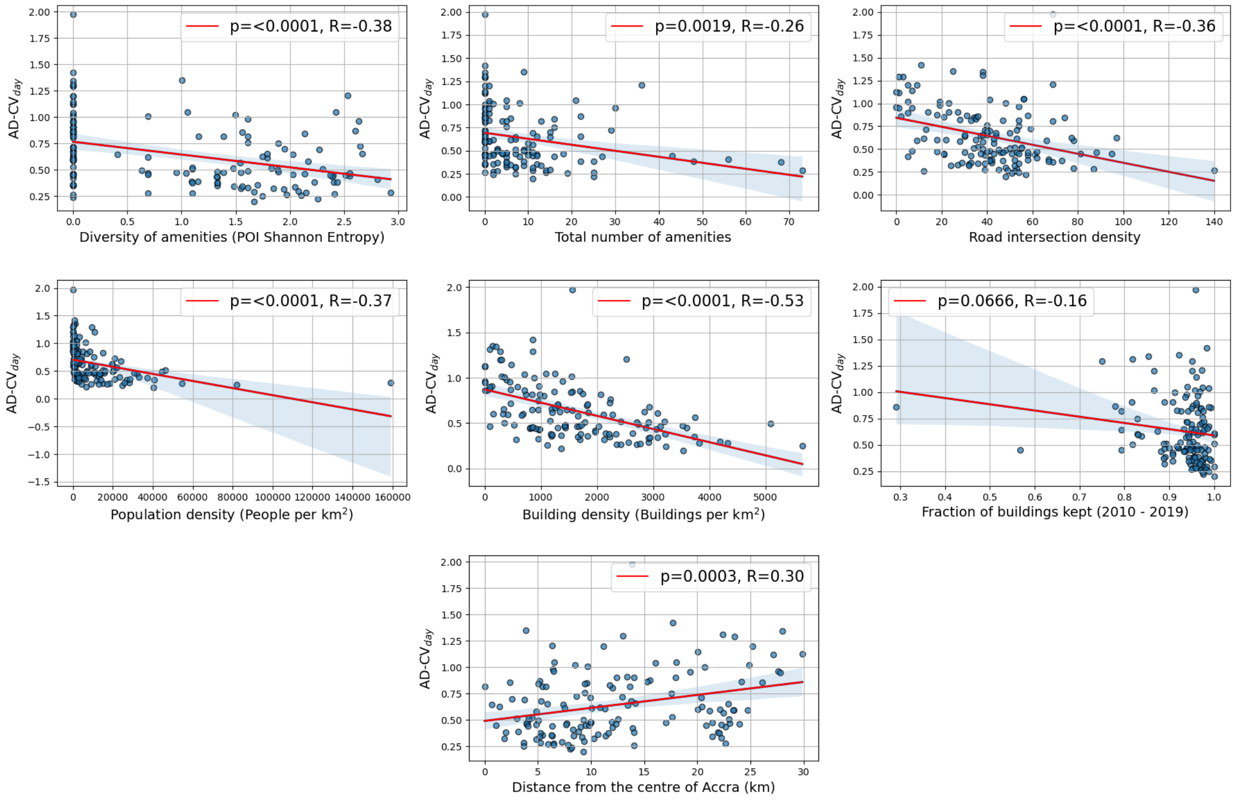

## Appendix Figure E: Single variable linear regressions of AD-$CV_{night}$ against generators of diversity

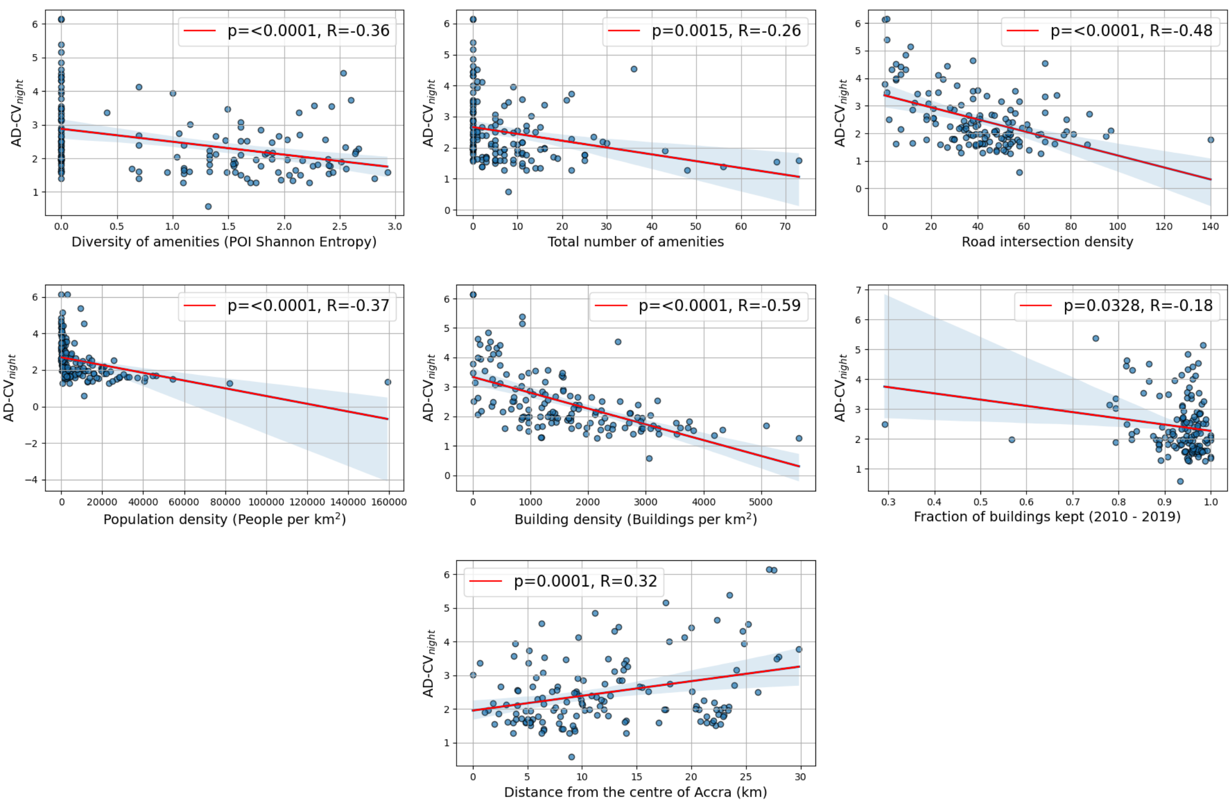

**Appendix Figure F: Comparison of coefficients from multivariate linear regression of AD-$CV_{day}$ and AD-$CV_{night}$ against generators of diversity**

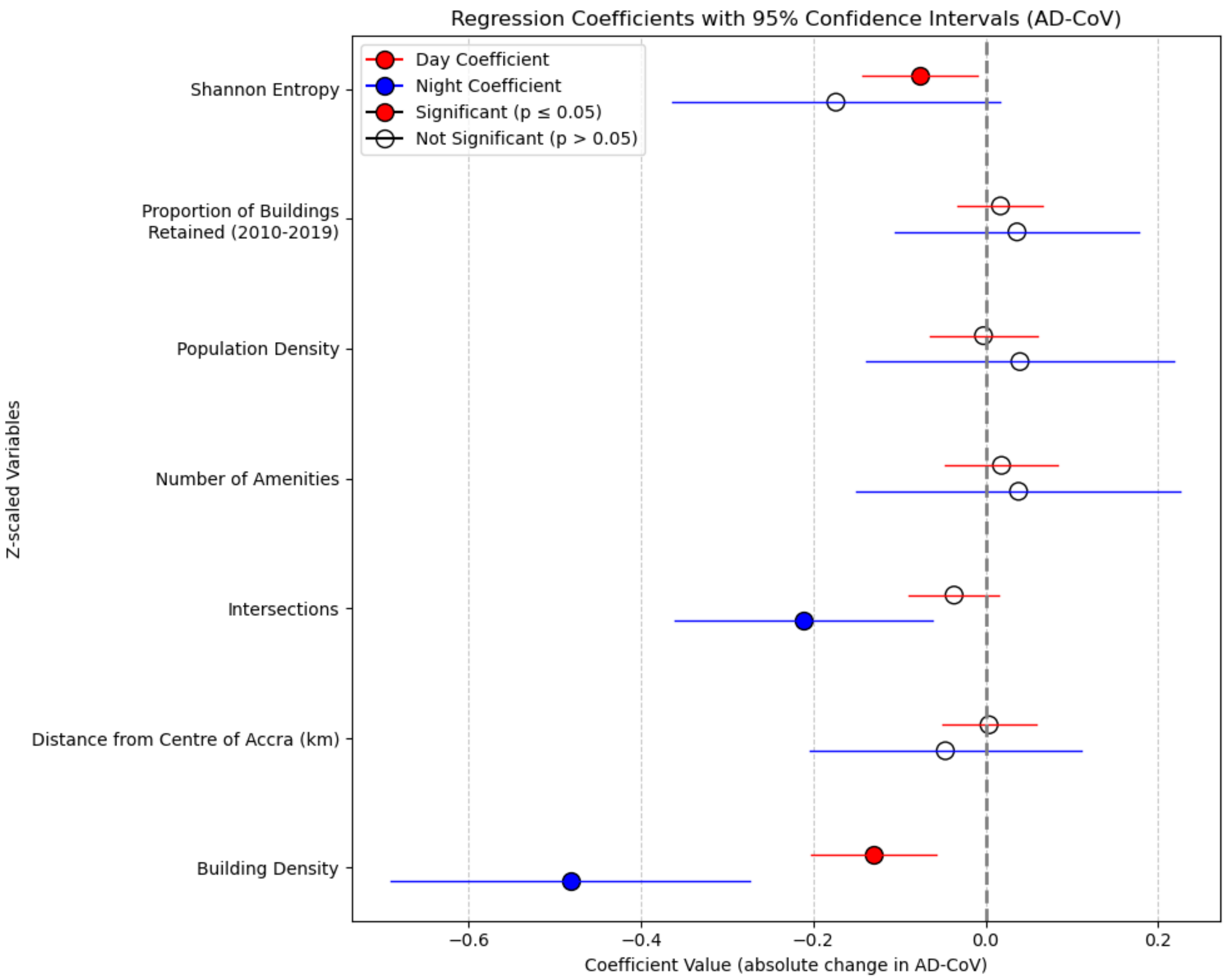

# Appendix Figure G: Spatial plots of AD-CV for pedestrians and vehicles

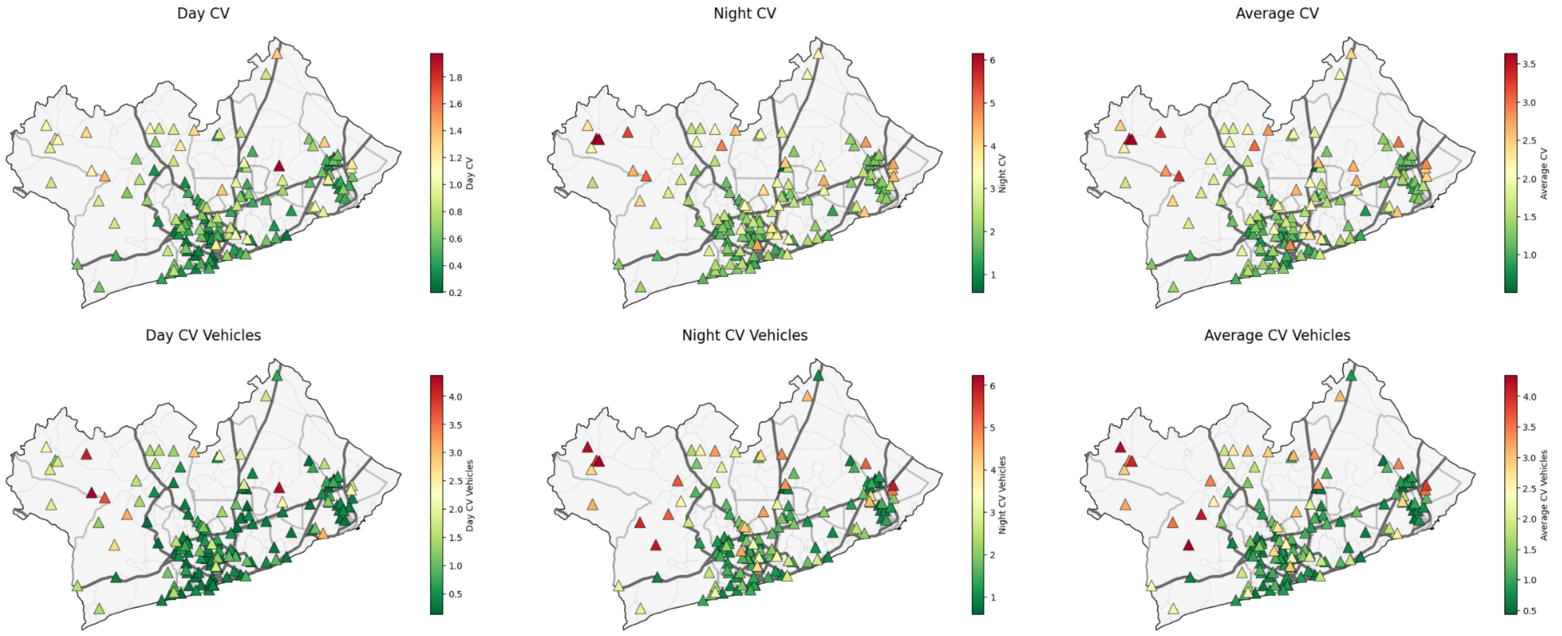

## Appendix Figure H: Single variable linear regressions of AD-CV$_{vehicles}$ against generators of diversity

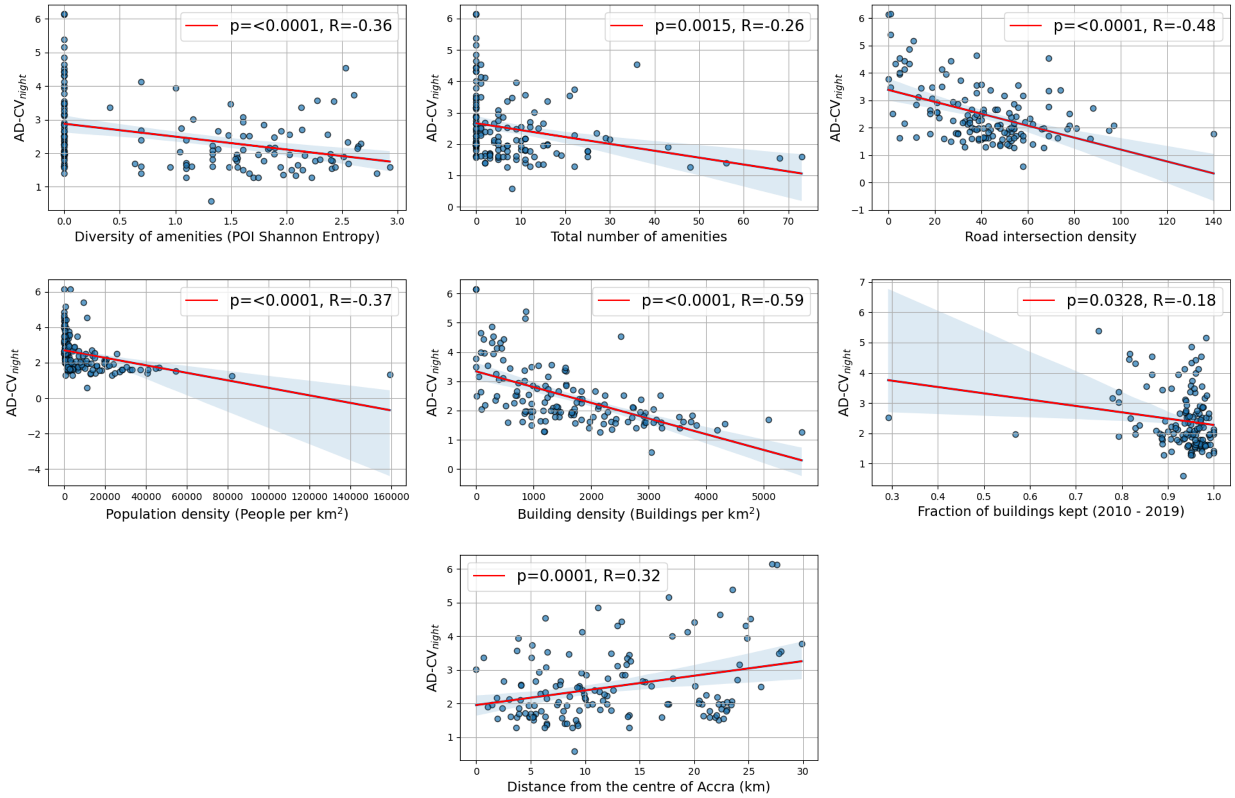

**Appendix Table A: Comparison of our study with previous studies comparing urban vitality with generators of diversity.**

| Study | Location | Urban vitality measure | Mixed-use measure | Block density measure | Building age measure | Building/population density measure(s) | Modelling approach |
|---|---|---|---|---|---|---|---|
| Sung et al., 2013 | Seoul, South Korea | Number of pedestrians (survey data). | Shannon entropy of land use categories (administrative data) | Intersection density (administrative data) | Year of construction (administrative data) | Number of buildings per normalised area (administrative data). | Multiple linear regression |
| Jacobs-Crisioni et al., 2014 | Amsterdam, Netherlands | Density of phone calls (mobile phone data) | Co-location of different building uses (building footprint data) | N/A | N/A | Fraction of areas occupied by building types (building footprint data) | Multiple linear spatial error model |
| Sung et al., 2015 | Seoul, South Korea | Fraction of pedestrians walking at given time of day (survey data) | Shannon entropy of land use categories (administrative data) | Intersection density (administrative data) | Year of construction (administrative data) | Population density and building density (segregated by type) (administrative data) | Multilevel binomial logistic model |
| De Nadai et al., 2016 | Bologna, Florence, Milan, Palermo, Rome, and Turin; Italy | Mobile Internet activity density (number of internet connections) (mobile phone data) | Land-use cover (remote-sensing derived estimates) and Foursquare location data. | Block area and intersection density (administrative data) | Age of construction (banded) (administrative data) | Number of residents and buildings (administrative data) | Multiple linear regression |
| Yue et al., 2017 | Shenzhen, China | Number of phone users (mobile phone data) | Shannon entry and Hill numbers of POI categories (Map database) | N/A | N/A | Population density (administrative data) | Multiple linear regression |
| Delclòs-Alió et al., 2019 | Barcelona, Spain | Geotagged locations from 241 volunteers (GPS data) | Shannon entropy (building footprint data) | Block size (building footprint data) | Year of construction (building footprint data) | Population density (census) | Descriptive differences between population groups |

| Study | Location | Urban vitality measure | Mixed-use measure | Block density measure | Building age measure | Building/population density measure(s) | Modelling approach |
|---|---|---|---|---|---|---|---|
| Kang et al., 2021 | Wuhan, China | Density of phone calls (mobile phone data) and perception of vitality (survey data) | Shannon entropy, richness and Simpson index (administrative data) | N/A | N/A | Mobile phone activity (mobile phone data) | Multiple linear regression |
| Scepanovic et al., 2021 | Bologna, Florence, Milan, Palermo, Rome, and Turin; Italy | Mobile Internet activity density (number of internet connections) (mobile phone data) | Land-use cover (remote-sensing derived estimates) | Block area and intersection density (administrative data) | Age of construction (banded) (administrative data) | Number of residents and buildings (administrative data) | Multiple linear regression, ElasticNet, SVM Regression and Gradient Boosted Trees |
| Gómez-Varo et al., 2022 | Barcelona, Spain | N/A (not measured as an independent outcome) | Shannon entropy and normalise difference in residential vs non-residential (administrative data) | Block size (building footprint data) | Year of construction (administrative data) | Population density (administrative data) | Composite index construction (no regression; descriptive metrics) |
| **Our study (2024)** | Accra, Ghana | Coefficient of variance in hourly footfall (detected from images) | Shannon entropy of points of interest within buffer (OSM) | Number of intersections in buffer (OSM) | Proportion of building stock (2019) added/ removed since 2010 (remote sensing estimate) | Building footprints (remote-sensing derived) and Ghana Statistical Service Census (2010) | Multiple linear regression |

**Appendix Table B: Total counts of each amenity category detected across all sites.**

| Amenity category | Total |
|---|---|
| School | 102 |
| Christian place of worship | 95 |
| Bank | 90 |
| Restaurant | 74 |
| Pitch | 59 |
| Hotel | 59 |
| Pharmacy | 45 |
| DIY shop | 44 |
| Bar | 31 |
| Pub | 29 |
| Convenience shop | 27 |
| Fast food outlet | 26 |
| Hairdresser | 25 |
| Kiosk | 23 |
| Toilet | 23 |
| ATM | 16 |
| Comms tower | 16 |
| Swimming pool | 15 |
| Post office | 15 |
| Guest house | 15 |
| Mobile phone shop | 14 |
| Clothes shop | 14 |
| Embassy | 14 |
| Hospital | 13 |
| Police station | 13 |
| Golf course | 13 |
| Shelter | 12 |
| Muslim place of worship | 11 |
| Car dealership | 9 |
| Greengrocer | 9 |
| Computer shop | 9 |
| Supermarket | 8 |
| Tower | 8 |
| Kindergarten | 8 |
| Car wash | 8 |
| Book shop | 8 |
| Night club | 7 |
| Park | 7 |
| Wastebasket | 7 |
| Doctor's practice | 7 |
| Beauty shop | 7 |
| Stadium | 6 |
| Gift shop | 6 |
| Furniture shop | 6 |
| College | 6 |
| Hostel | 6 |
| Beverage shop | 5 |
| Bakery | 5 |
| Toy shop | 4 |
| Sports centre | 4 |
| Playground | 4 |
| Monument | 4 |
| Chemist | 4 |
| University | 4 |
| Graveyard | 4 |
| Attraction | 3 |
| Butcher | 3 |
| Recycling | 3 |
| Stationery shop | 3 |
| Cafe | 3 |
| Dentist | 3 |
| Anglican place of worship | 3 |
| Optician | 3 |
| Mall | 2 |
| Theatre | 2 |
| Fire station | 2 |
| Jeweller | 2 |
| Bench | 2 |
| Castle | 2 |
| Department store | 2 |
| Courthouse | 2 |
| Track | 2 |
| Veterinary clinic | 2 |
| Cinema | 2 |
| Methodist place of worship | 2 |
| Christian orthodox place of worship | 1 |
| Library | 1 |
| Wastewater plant | 1 |
| Sports shop | 1 |
| Car rental | 1 |
| Shoe shop | 1 |
| Travel agent | 1 |
| Nursing home | 1 |
| Town hall | 1 |
| Fountain | 1 |
| Telephone | 1 |
| Arts centre | 1 |

**Appendix Table C: Performance of building classification algorithm**

| 2010 images algorithm | Recall | Precision | F1 |
|---|---|---|---|
| **Total** | 81.00% | 81.00% | 80.00% |
| Building | 90.00% | 91.00% | 90.00% |
| Empty land | 85.00% | 65.00% | 74.00% |
| Incomplete building | 84.00% | 92.00% | 88.00% |
| Partial rooftop | 21.00% | 52.00% | 30.00% |

| 2019 images algorithm | Recall | Precision | F1 |
|---|---|---|---|
| **Total** | 83.00% | 84.00% | 82.00% |
| Building | 91.00% | 92.00% | 92.00% |
| Empty land | 72.00% | 43.00% | 54.00% |
| Incomplete building | 88.00% | 89.00% | 88.00% |
| Partial rooftop | 22.00% | 63.00% | 33.00% |

The performance of our Resnet-50 image classification algorithm, developed in a separate study (Nathvani and D., 2024), applied to tiles of very high resolution (0.3m/pixel) Maxar satellite images, where each tile corresponds to a building footprint (Maxar, 2020). Separate classification algorithms were trained for 2010 and 2019 satellite images based on a hand-labelled dataset of 5500 images (split 60/20/20 for training, validation and testing), described elsewhere, and applied to all building footprint locations across both years. For this study all instances of "Incomplete building" and "Partial rooftop" are discarded.

## References


Clark, S.N., Alli, A.S., Brauer, M., Ezzati, M., Baumgartner, J., Toledano, M.B., Hughes, A.F., Nimo, J., Bedford Moses, J., Terkpertey, S., Vallarino, J., Agyei-Mensah, S., Agyemang, E., Nathvani, R., Muller, E., Bennett, J., Wang, J., Beddows, A., Kelly, F., Barratt, B., Beevers, S., Arku, R.E., 2020. High-resolution spatiotemporal measurement of air and environmental noise pollution in Sub-Saharan African cities: Pathways to Equitable Health Cities Study protocol for Accra, Ghana. BMJ Open 10. https://doi.org/10.1136/bmjopen-2019-035798

De Nadai, M., Staiano, J., Larcher, R., Sebe, N., Quercia, D., Lepri, B., 2016. The Death and Life of Great Italian Cities: A Mobile Phone Data Perspective, in: Proceedings of the 25th International Conference on World Wide Web. Presented at the WWW '16: 25th International World Wide Web Conference, International World Wide Web Conferences Steering Committee, Montréal Québec Canada, pp. 413–423. https://doi.org/10.1145/2872427.2883084

Delclòs-Alió, X., Gutiérrez, A., Miralles-Guasch, C., 2019. The urban vitality conditions of Jane Jacobs in Barcelona: Residential and smartphone-based tracking measurements of the built environment in a Mediterranean metropolis. Cities 86, 220–228. https://doi.org/10.1016/j.cities.2018.09.021

Gómez-Varo, I., Delclòs-Alió, X., Miralles-Guasch, C., 2022. Jane Jacobs reloaded: A contemporary operationalization of urban vitality in a district in Barcelona. Cities 123, 103565. https://doi.org/10.1016/j.cities.2022.103565

Jacobs-Crisioni, C., Rietveld, P., Koomen, E., Tranos, E., 2014. Evaluating the Impact of Land-Use Density and Mix on Spatiotemporal Urban Activity Patterns: An Exploratory Study Using Mobile Phone Data. Environ. Plan. Econ. Space 46, 2769–2785. https://doi.org/10.1068/a130309p

He, K., Zhang, X., Ren, S., Sun, J., 2016. Deep Residual Learning for Image Recognition, in: 2016 IEEE Conference on Computer Vision and Pattern Recognition (CVPR). Presented at the 2016 IEEE Conference on Computer Vision and Pattern Recognition (CVPR), pp. 770–778. https://doi.org/10.1109/CVPR.2016.90

Kang, C., Fan, D., Jiao, H., 2021. Validating activity, time, and space diversity as essential components of urban vitality. Environ. Plan. B Urban Anal. City Sci. 48, 1180–1197. https://doi.org/10.1177/2399808320919771

Maxar, 2020. Maxar Technologies. 2020. COVID-19 Open data program.

Microsoft, 2023. Worldwide building footprints derived from satellite imagery.

Nathvani, R., Clark, S.N., Muller, E., Alli, A.S., Bennett, J.E., Nimo, J., Moses, J.B., Baah, S., Metzler, A.B., Brauer, M., Suel, E., Hughes, A.F., Rashid, T., Gemmell, E., Moulds, S., Baumgartner, J., Toledano, M., Agyemang, E., Owusu, G., Agyei-Mensah, S., Arku, R.E., Ezzati, M., 2022. Characterisation of urban environment and activity across space and time using street images and deep learning in Accra. Sci. Rep. 12, 20470. https://doi.org/10.1038/s41598-022-24474-1

Nathvani, R., D., V., 2024. Neighbourhood level changes in housing across Accra, Ghana between 2010 - 2019 from high resolution remote sensing. Prep. Submiss.

Scepanovic, S., Joglekar, S., Law, S., Quercia, D., 2021. Jane Jacobs in the Sky: Predicting Urban Vitality with Open Satellite Data. Proc. ACM Hum.-Comput. Interact. 5, 48:1-48:25. https://doi.org/10.1145/3449257

Sirko, W., Kashubin, S., Ritter, M., Annkah, A., Bouchareb, Y.S.E., Dauphin, Y., Keysers, D., Neumann, M., Cisse, M., Quinn, J., 2021. Continental-Scale Building Detection from High Resolution Satellite Imagery. ArXiv210712283 Cs.

Sung, H., Lee, S., Cheon, S., 2015. Operationalizing Jane Jacobs's Urban Design Theory: Empirical Verification from the Great City of Seoul, Korea. J. Plan. Educ. Res. 35, 117–130. https://doi.org/10.1177/0739456x14568021

Sung, H.-G., Go, D.-H., Choi, C.G., 2013. Evidence of Jacobs's street life in the great Seoul city: Identifying the association of physical environment with walking activity on streets. Cities 35, 164–173. https://doi.org/10.1016/j.cities.2013.07.010

Yue, Y., Zhuang, Y., Yeh, A.G.O., Xie, J.-Y., Ma, C.-L., Li, Q.-Q., 2017. Measurements of POI-based mixed use and their relationships with neighbourhood vibrancy. Int. J. Geogr. Inf. Sci. 31, 658–675. https://doi.org/10.1080/13658816.2016.1220561
